# Predicting the clinical outcome of melanoma using an immune-related gene pairs signature

**DOI:** 10.1371/journal.pone.0240331

**Published:** 2020-10-08

**Authors:** Liangliang Meng, Xiaoxi He, Xiao Zhang, Xiaobo Zhang, Yingtian Wei, Bin Wu, Wei Li, Jing Li, Yueyong Xiao

**Affiliations:** 1 Medical School of Chinese PLA, Beijing, China; 2 Department of Radiology, The First Medical Centre, Chinese PLA General Hospital, Beijing, China; 3 Department of Radiology, Chinese PAP Beijing Corps Hospital, Beijing, China; 4 Department of Radiology, Tianjin Medical University Cancer Institute and Hospital, National Clinical Research Center for Cancer, Tianjin, China; Universidade de Sao Paulo, BRAZIL

## Abstract

**Objective:**

Melanoma is rare but dangerous skin cancer, and it can spread rather quickly in the advanced stages of the tumor. Abundant evidence suggests the relationship between tumor development and progression and the immune system. A robust gene risk model could provide an accurate prediction of clinical outcomes. The present study aimed to explore a robust signature of immune-related gene pairs (IRGPs) for estimating overall survival (OS) in malignant melanoma.

**Methods:**

Clinical and genetic data of skin cutaneous melanoma (SKCM) patients from The Cancer Genome Atlas (TCGA) was performed as a training dataset to identify candidate IRGPs for the prognosis of melanoma. Two independent datasets from the Gene Expression Omnibus (GEO) database (GSE65904) and TCGA dataset (TCGA-UVM) were selected for external validation. Univariate and multivariate Cox regression analyses were then performed to explore the prognostic power of the IRGPs signature and other clinical factors. CIBERSORTx was applied to estimate the fractions of infiltrated immune cells in bulk tumor tissues.

**Results:**

A signature consisted of 33 IRGPs was established which was significantly associated with patients’ survival in the TCGA-SKCM dataset (P = 2.0×10^−16^, Hazard Ratio (HR) = 4.220 (2.909 to 6.122)). We found the IRGPs signature exhibited an independent prognostic factor in all the three independent cohorts in both the univariate and multivariate Cox analysis (P<0.01). The prognostic efficacy of the signature remained unaffected regardless of whether *BRAF* or *NRAS* was mutated. As expected, the results were verified in the GSE65904 dataset and the TCGA-UVM dataset. We found an apparent shorter OS in patients of the high-risk group in the GSE65904 dataset (P = 2.1×10^−3^; HR = 1.988 (1.309 to 3.020)). The trend in the results of the survival analysis in TCGA-UVM was as we expected, but the result was not statistically significant (P = 0.117, HR = 4.263 (1.407 to 12.91)). CD8 T cells, activated dendritic cells (DCs), regulatory T cells (Tregs), and activated CD4 memory T cells presented a significantly lower fraction in the high-risk group in the TCGA-SKCM dataset(P <0.01).

**Conclusion:**

The results of the present study support the IRGPs signature as a promising marker for prognosis prediction in melanoma.

## Introduction

Melanoma is a kind of malignant tumor that originates from melanocytes [1]. If not found and treated at an early stage, melanoma may rapidly spread to other organs of the whole body, which may lead to approximately 10,000 deaths in the USA each year [2]. Primary tumors are more commonly located in the lower extremities. For primary malignant melanoma that has not metastasized early, surgical resection is the most effective method, and more than 90% of patients have more than five years of survival [3]. Once metastasis occurs, surgical treatment will not help. For patients with more extensive melanoma, neighboring lymph nodes will be examined to determine whether metastasis has occurred. Moreover, the degree of improvement in radiotherapy and chemotherapy is minimal, so the mortality rate is very high in advanced melanoma [4].

As an immunogenic tumor, melanoma can overcome the effects of the immune system on it by producing inhibitory growth factors, cytokines, etc. in the tumor microenvironment [5]. According to the existing evidence, the immune system has an extensive influence on tumorigenesis and development. Experts around the world have been exploring new treatments for melanoma, led by immunotherapy and targeted therapy, with promising results [2, 6]. *BRAF* mutations are found in the genes of about half of melanoma patients, with younger patients making up the majority. And targeted *BRAF* inhibitors have been shown to significantly improve patients' physical condition and survival expectations [6]. In recent years, targeted therapies for melanoma have been converted from conventional *BRAF* inhibitors to combination therapy with both *MEK* and *BRAF* inhibitors [7, 8]. Immune-checkpoint inhibitors are currently considered as the most promising strategies in tumor immunotherapy. Studies have found that the combined use of programmed cell death-1 (*PD-1*) and cytotoxic T-lymphocyte antigen-4 (*CTLA-4*) immune checkpoint inhibitors significantly enhance the anti-tumor immune response of CD8+ T cells and inhibit regulatory T cell function in tumor treatment compared to their use alone [9–11].

These show that the development and treatment of melanoma are very closely related to immunity. There have also been many studies that have explored the relationship between immune genes and tumor prognosis and have found some valuable markers. Sheng et al. constructed an immune risk score (IRS), which is significantly associated with melanoma metastasis [1]. Recently, Cursons et al. developed a new method to explore nature killer (NK) cell infiltration in tumor tissue, and they found that metastatic skin melanoma patients who had evidence of NK cell infiltration in their tumors had an increased survival rate [12]. Chen et al. explored a signature consisting of four long non-coding RNAs (LncRNAs) that stratify the prognostic risk of melanoma patients, which may help improve prognosis in early-stage patients [13]. However, these signatures were not applied to the clinical application due to the small sample sizes and inadequate validation datasets. Also, the diversity of data from different sequencing platforms and the heterogeneity of tumors affect the integration and analysis of a large amount of gene expression data. Standardization of data across platforms is also the focus and difficulty of the analysis. Recently, a new approach has been developed based on the relative sequencing of gene expression levels that overcomes the shortcomings of traditional gene expression data processing and has yielded stable and reliable results in a variety of studies [14–17].

In our research, the expression levels of a range of immune-related genes in each tumor sample were compared in pairs, ultimately generating a score for each immune gene pair [14, 17]. Scoring for this immunogene pair-based approach is based entirely on gene expression profiles within a single tumor sample. It does not need to be normalized across samples to account for differences between multiple samples or sequencing platforms [17]. We used skin cutaneous melanoma (SKCM) program from the Cancer Genome Atlas (TCGA) RNA-seq dataset to construct an immune-related gene pair signature and to validate it by using the Gene Expression Omnibus (GEO) dataset (GSE65904) and uveal melanoma dataset (TCGA-UVM). Subsequently, we confirmed the efficacy of this immunomarker in predicting tumor prognosis by comparing it with other clinicopathological information. The relationship between these prognostic immune gene pairs and tumor-infiltration lymphocyte cell content was further explored.

## Material and methods

### Data sources of melanoma

The HTSeq-FPKM RNA-seq expression data and clinical data of 471 skin cutaneous melanoma patients were retrieved from The TCGA-SKCM dataset (https://portal.gdc.cancer.gov). GSE65904 dataset retrieved from the GEO database (http://www.ncbi.nlm.nih.gov/geo), and the uveal melanoma dataset (TCGA-UVM) retrieved from the TCGA database with corresponding survival information were recruited for external validation. The GSE65904 dataset was published in April 2015 and was based on the GPL10558 platform. Resected tumors from 214 unique melanoma samples were profiled on gene expression arrays. There were 80 patients in the TCGA-UVM dataset with clinical information and tumor tissue expression data. Patients with overall survival time (OS) less than one month or missing survival information were excluded from the study. In total, 639 cases were recruited and analyzed in the present study, of which 378 patients in the TCGA-SKCM group, 75 in the TCGA-UVM group, and 186 patients were in the GEO group. The clinical and pathological data of all included patients are shown in S1 Table.

### Gene expression data processing

The RNA-seq expression data was HTSeq-FPKM type. The expression profile data for each gene was converted to the corresponding gene symbol from the probe level according to the annotation file. No further standardization of the expressed data is required. If the patient has multiple samples, take the average expression value of each gene to represent the gene expression level of the patient. If there are multiple probes for a single gene, the average expression value will be taken as the expression level for that gene.

### Modeling of the immune-related gene pairs (IRGPs) signature

We downloaded a total of 1811 immune-related genes (IRGs) for constructing a prognostic signature from the ImmPort database (https://immport.niaid.nih.gov). IRGs include interleukins, interferons, cytokines, cytokine receptors, chemokines, chemokine receptors, natural killer cells, and genes related to the T-cell receptor signaling pathway, and others. A total of 1646 immune-related genes measured by both TCGA and GEO datasets platforms were selected for further analysis. The specific method of constructing the prognostic model is as described in the previous study [17]. Briefly, we performed a pairwise comparison to obtain a score for each gene pair between the gene expression value within each sample in the TCGA cohort. The score of a specific gene pair was set to one when the expression level of the first gene was higher than the other; otherwise was zero. We would discard the gene pair if more than 90% of the score of a gene pair were identity in the samples. First, we used both Cox and Kaplan-Meier methods to analyze the scoring and survival data of immune gene pairs to screen out the immune gene pairs that were significantly correlated with prognosis, and a total of 187 prognosis-correlated gene pairs were screened out (p-value < 0.0001). Then we used the lasso regression model with 1000 random cycles to eliminate highly correlated gene pairs and retain the most informative and least number of gene pairs as models. Finally, 33 pairs of genes were retained as a prognostic-related signature. The risk score for each patient was obtained based on the model we constructed. We stratified patients into low- and high-risk groups using the most appropriate cut-off of the IRGPs score. We used the "survivalROC" R package (Using R package "survivalROC", version 1.0.3) to obtain the optimal cut-off value by the time-dependent receiver operating characteristic (ROC) curve analysis at three years for OS in the TCGA dataset. We assigned a cut-off value at the point in the ROC curve with the maximum sum of sensitivity and specificity.

### Prognostic value of IRPGs in the TCGA-SKCM cohort

Survival analysis by the log-rank test was performed between the different immune risk groups. Subsequently, both univariate and multivariate Cox proportional hazards regression analyses of the risk factor and other clinical factors for the OS were performed in the TCGA cohort. We queried the cBioPortal website(https://www.cbioportal.org/) for mutations in multiple related genes in the TCGA dataset, with the highest number of patients with mutations in *BRAF* and *NRAS*. Of the 378 patients included in the analysis, the *BRAF* gene experienced mutations in 156 patients, and the *NRAS* gene experienced mutations in 87 patients. Of these 156 patients who experienced BRAF mutations, all mutation types were missense mutations, including 134 patients with BRAF V600E mutations, five patients with BRAF K601E mutations, three patients with BRAF G466E mutations, and 14 patients with other uncommon mutations. Of the 87 patients who experienced NRAS mutations, all mutation types were missense mutations except for one case with an X37 splice site mutation, of which 39 were NRAS Q61R mutations, 25 were Q61K mutations, 9 were Q61L mutations, 5 were Q61H mutations, and eight other rare mutations. And we then stratified the dataset into different sub-datasets based on *BRAF* and *NRAS* mutation, to verify whether these factors affect the test efficacy of the prognostic signature. Besides, to explore the relationship between *BRAF* or *NRAS* mutation status and the risk scores we obtained, we then divided patients into two groups based on whether they had *BRAF* or *NRAS* mutations and compared the difference in risk scores between the two groups.

### Dataset validation of the IRGPs signature

To further prove the prognostic merit of the IRGPs signature in different cohorts, we applied the IRGPs signature to two independent cohorts from the GEO database (GSE65904) and TCGA database (TCGA-UVM) for external validation. These datasets were stratified into high- and low-risk groups according to each patient's risk score, and survival analyses were performed separately. We also performed the same univariate and multivariate analysis as in the TCGA cohort.

### Estimation of immune cell abundance in tumor tissue

To analyze whether there were differences in the immune cell abundance of tumor tissue in different risk groups, we used CIBERSORTx (https://cibersortx.stanford.edu/) to evaluate the relative abundance of predefined cell types in mixed solid tissues. Normalized RNA-seq FPKM gene expression data from tumor tissues in the TCGA-SKCM dataset were used for this analysis. [18]. We used the default LM22 leukocyte gene signature matrix from the CIBERSORTx website. LM22 contains 547 genes distinguishing 22 types of immune-related cells. Disabling quantile normalization was checked. We set the number of permutations to 1000 for robust analyses. Then CIBERSORTx enumerated the relative proportions of the 22 infiltrating immune cells, including B cells, dendritic cells (DCs), T cells, natural killer cells, macrophages, and others. The Wilcoxon rank-sum test with continuity correction was applied to evaluate cell proportions between high- and low-risk groups using an adjusted P-threshold.

### Statistical analyses

Statistical analyses were mainly performed on R software (version 3.6.3, www.r-project.org). Survival analyses were performed using the 'survival' package (version 3.1–11) with the Kaplan-Meier method. We used the student's two-sample t-test or Wilcoxon rank-sum test to compare the continuous variables. For comparisons of multiple different variables (e.g., Cell abundance) between two groups, we used a false discovery rate (FDR) method to correct for multiple comparisons of the results. The 'survival' package also calculated the root mean square (RMS) curve and time ratio. For all analyses, the statistical threshold was set to P-value<0.05.

### The Kyoto Encyclopedia of Genes and Genomes (KEGG) pathway and Gene set enrichment analyses (GSEA)

We performed the KEGG pathway analysis for the prognostic immune signature genes using the "clusterProfiler" R package (version 3.12.0) [19]. We used P-value < 0.05 as the threshold for KEGG enrichment analysis. GSEA is used to assess the distribution trends of genes in a predefined set of genes in a gene set sequenced for phenotypic relevance and thus to determine their contribution to the phenotype [20]. We applied the GSEA software (Version 4.0.3, http://software.broadinstitute.org/gsea/) with 1,000 phenotype permutations for GSEA enrichment analysis. The threshold of statistically significant gene sets was set to a nominal P-value < 0.05 with FDR adjusted P-value < 0.25. We classified the patients into two groups according to their risk values. We then performed a GSEA analysis to compare whether there were pathways of differential enrichment between the two groups. MSigDB Hallmark gene sets (version 7.1, https://www.gsea-msigdb.org/gsea/downloads.jsp) was applied in the GSEA analysis.

## Results

### Construction of the IRGPs signature

**Fig 1:**
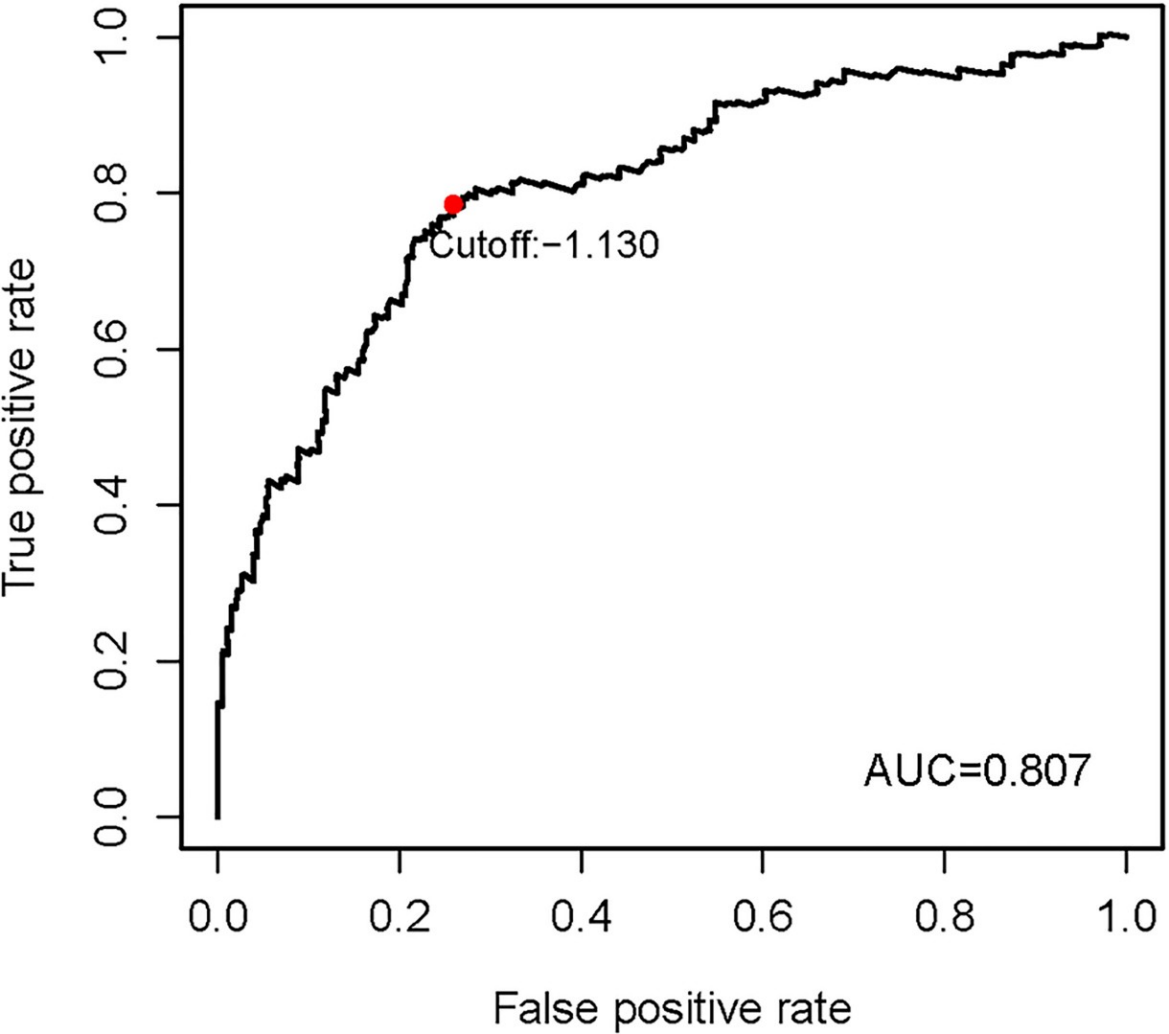
The optimal cut-off value of the IRGPs risk-score obtained by the time-dependent ROC curve analysis. Abbreviations: IRGPs, immune-related gene pairs; ROC, receiver operating characteristic; AUC, area under curve.

**Fig 2:**
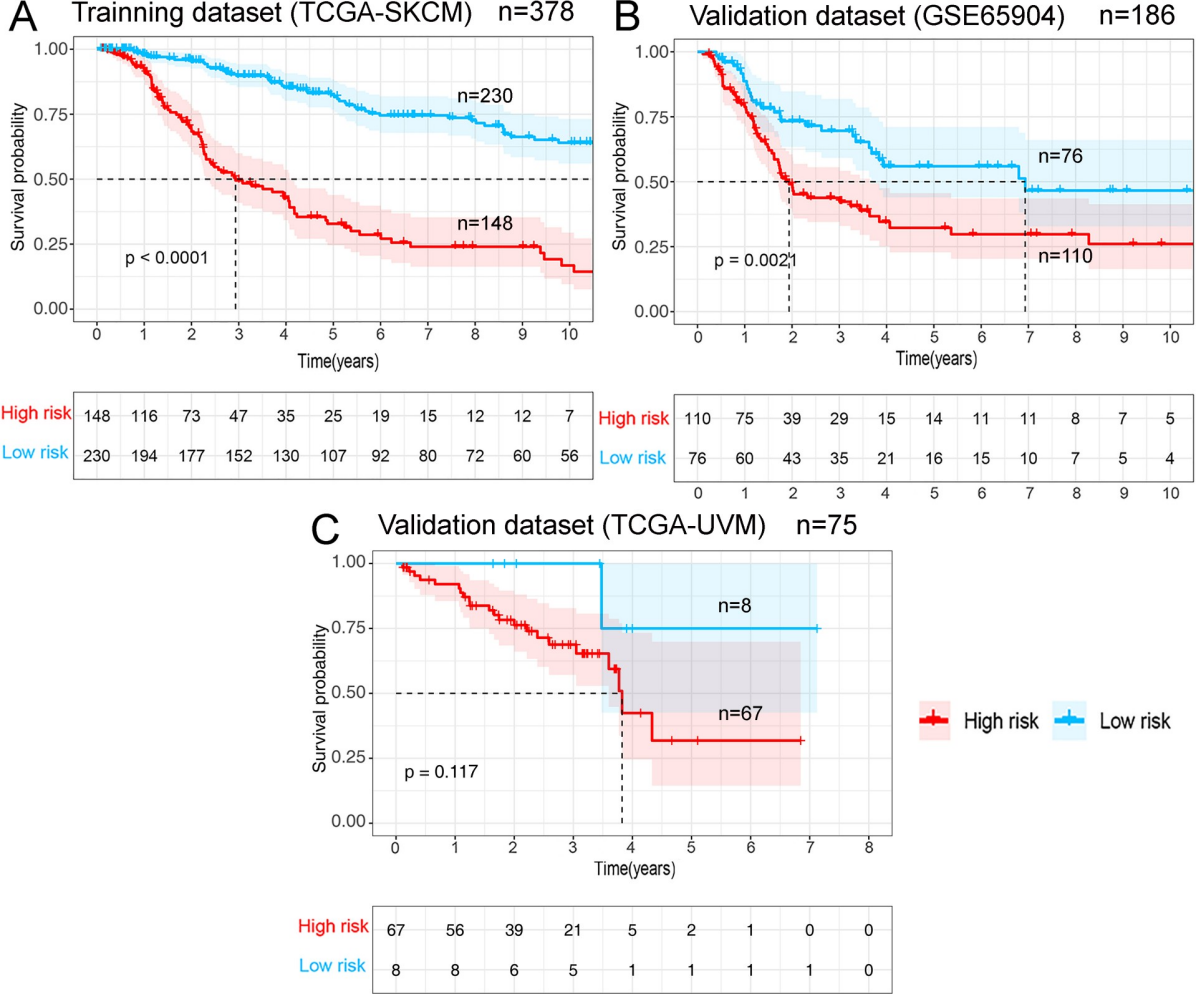
Survival curves for different risk groups. According to the optimal cut-off value, patients from different cohorts were stratified into the high- or low- risk group. Kaplan-Meier curves were used for survival analyses between different risk groups in different datasets: (A) TCGA-SKCM training dataset. (B) The GEO external validation cohort (GSE65904). (C) The TCGA-UVM external validation cohort.

**Fig 3:**
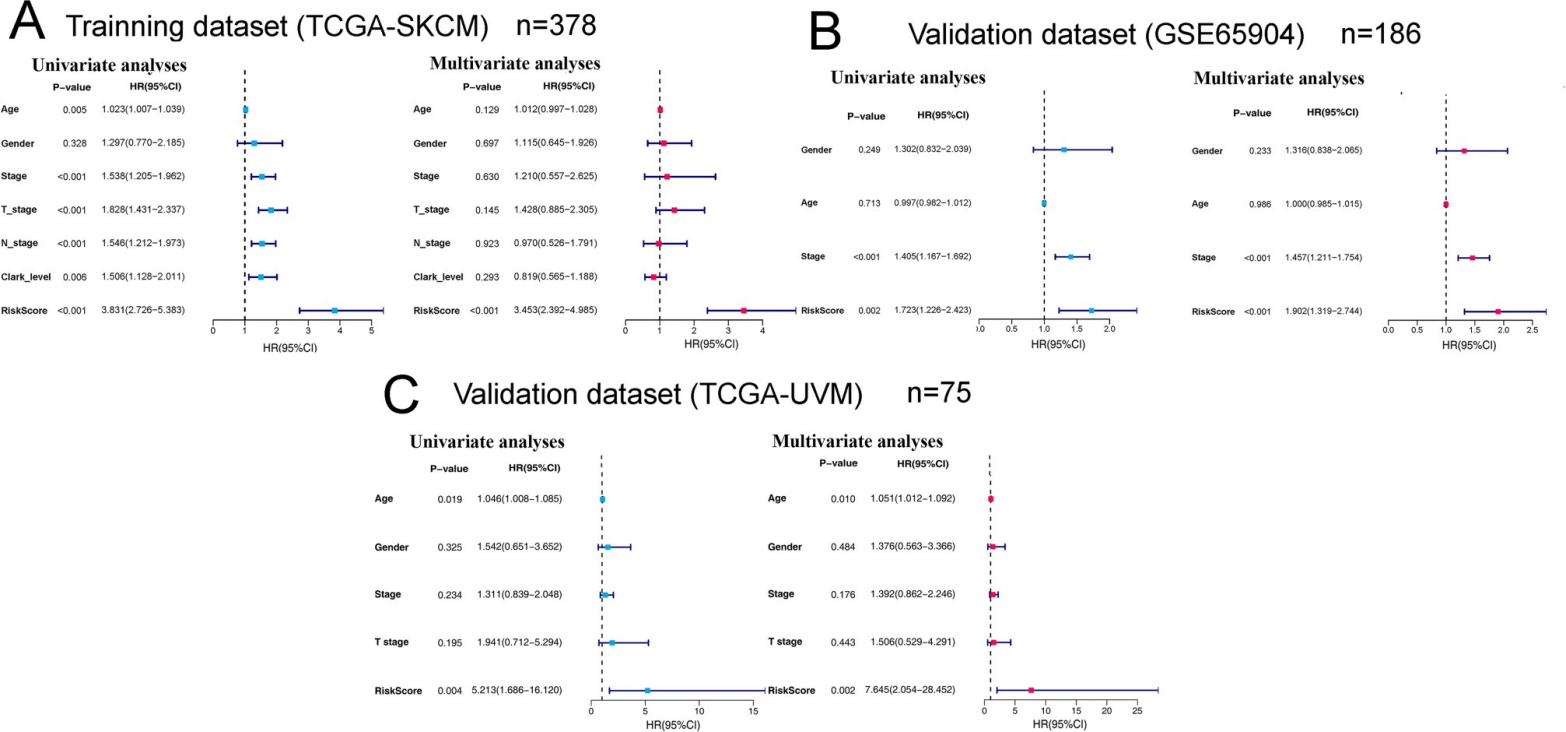
Forest plots of univariate and multivariate Cox regression analyses in different cohorts. (A) TCGA-SKCM training cohort (n = 378). (B)The GEO validation cohort (n = 186). (C) The TCGA-UVM external validation cohort (n = 75). HR, hazard ratio.

**Fig 4:**
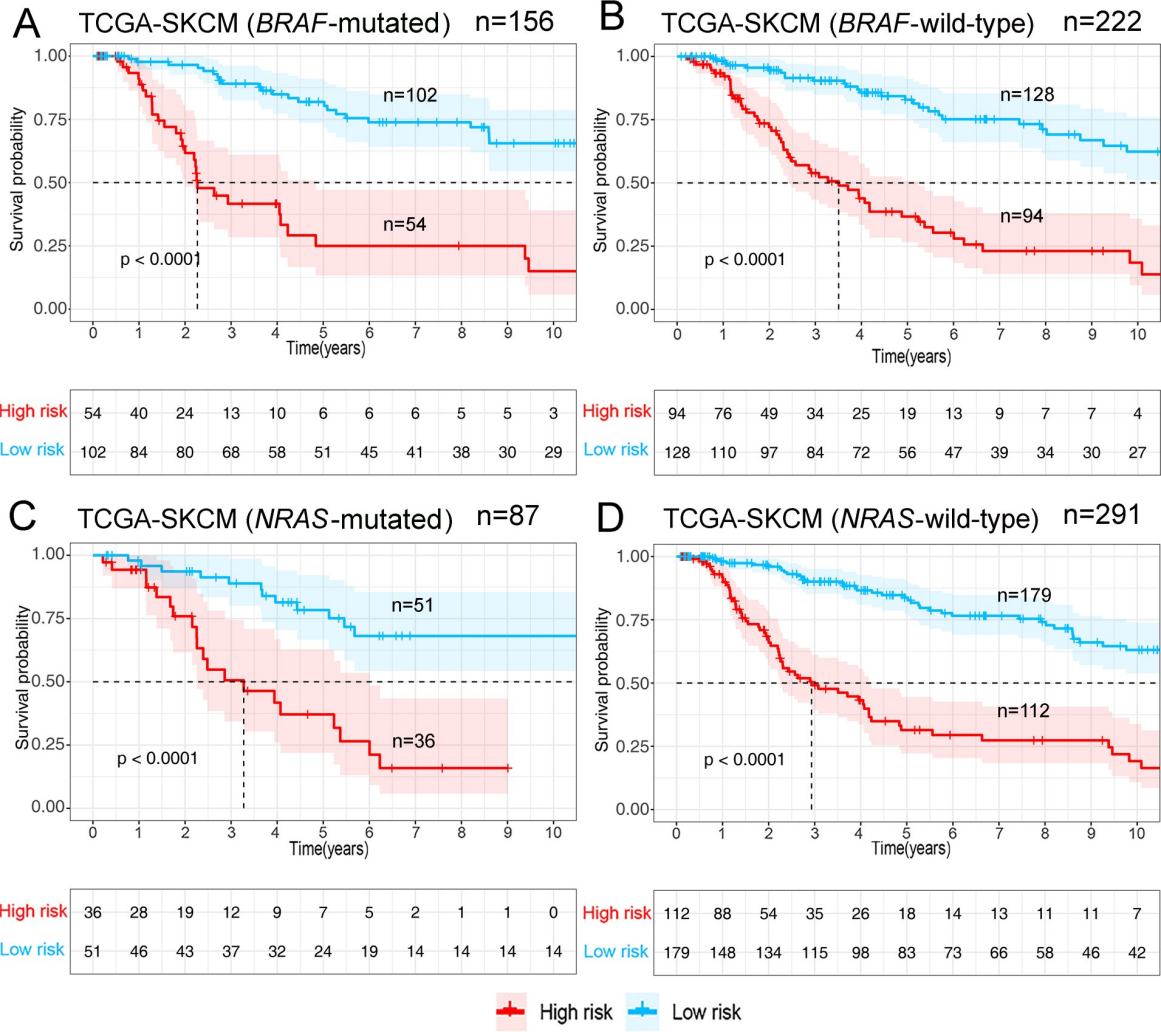
Survival analyses after grouping according to gene mutation status in the TCGA-SKCM dataset. (A) BRAF-mutated patients. (B) BRAF-wild-type patients. (C) NRAS-mutated patients. (D) NRAS-wild-type patients. All groupings, according to the genotypes, had no effect on the predictive validity of the markers.

Gene expression data of the TCGA-SKCM cohort was used as an exploratory dataset. Genes with an average expression greater than 0 and genes with median absolute deviation (MAD) >0.5 are included in the subsequent analysis. A total of 378 patients were recruited in the exploratory dataset. We applied a screened exploratory dataset to construct the survival model. 624 IRGs contained in both the exploratory set and the validation set were included in the model construction. The total number of gene pairs established was 32997. After rigorous screening to remove relatively small variation IRGPs (MAD = 0), only 187 candidate IRGPs were left for further study. Finally, a series of 33 IRGPs were recruited in the risk model using Lasso Cox proportional hazards regression from the TCGA cohort. Detailed information on the immune gene pair model can be found in Table 1. Using the model, we can calculate a risk score of IRGPs for each sample. The fittest cut-off value of the IRGPs risk score was set at −1.130 using a time-dependent ROC curve analysis. We then stratified the dataset into the high- or low- risk group according to the cut-off value (Fig 1). Significantly, compared to the low-risk group, the high-risk group in the exploratory TCGA cohort exhibited a poorer OS (P = 2.0×10^−16^, Hazard Ratio (HR) = 4.220 (2.909 to 6.122)) (Fig 2A and Table 2). Significant differences in age, pathologic tumor stage, T stage, N stage, and Clark level were shown between groups related to OS in the univariate Cox analysis (P<0.01). However, in the multivariate Cox, only the phenotype of IRGPs signature exhibited a robust independent prognostic factor (P<0.001) (see in Fig 3A and Table 2 for details). Besides, to analyze whether frequent genetic mutations affect the efficacy of the prognostic signature, we grouped the TCGA-SKCM dataset according to whether *BRAF*or *NRAS* was mutated, respectively. Consistent results were obtained for all four subsets of the dataset. Survival analyses in all groups showed the prognostic accuracy of the signature independent of whether these common genes were mutated or not (P<0.0001) (see in Fig 4). The superiority of the signature over other clinical factors as independent prognostic factors was also not affected by *BRAF* or *NRAS* mutation in both univariate and multivariate analyses (see in S1 Fig). Besides, we divided patients into two groups based on whether they had *BRAF* or *NRAS* mutations and compared the difference in risk scores between the two groups. Interestingly, we found a lower risk score in the *BRAF* mutation group (P = 0.03947), suggesting that patients may have a better prognosis than those in the non-mutation group. However, when analyzing the *NRAS* mutations, we did not find any difference in risk scores between the two groups (P = 0.1897).

**Table 1:** Prognostic IRGPs signature.

Gene-A	Full name-A	Gene-B	Full name-B	Coefficient
*HFE*	homeostatic iron regulator	*SLPI*	secretory leukocyte peptidase inhibitor	-0.03375
*HFE*	homeostatic iron regulator	*LHB*	luteinizing hormone subunit beta	-0.18429
*HLA-DQA2*	major histocompatibility complex, class II, DQ alpha 2	*SLPI*	secretory leukocyte peptidase inhibitor	-0.00242
*HLA-DQB1*	major histocompatibility complex, class II, DQ beta 1	*S100A8*	S100 calcium binding protein A8	-0.52645
*HSPA2*	heat shock protein family A (Hsp70) member 2	*APOBEC3G*	apolipoprotein B mRNA editing enzyme catalytic subunit 3G	0.00529
*MICB*	MHC class I polypeptide-related sequence B	*OAS1*	2'-5'-oligoadenylate synthetase 1	0.054884
*PSME1*	proteasome activator subunit 1	*IFITM1*	interferon-induced transmembrane protein 1	0.120809
*PI3*	peptidase inhibitor 3	*FGF1*	fibroblast growth factor 1	0.060244
*PI3*	peptidase inhibitor 3	*LCP2*	lymphocyte cytosolic protein 2	0.023869
*SLPI*	secretory leukocyte peptidase inhibitor	*KDR*	kinase insert domain receptor	0.24411
*CCL13*	C-C motif chemokine ligand 13	*FABP4*	fatty acid-binding protein 4	-0.13567
*CCL8*	C-C motif chemokine ligand 8	*STC1*	stanniocalcin 1	-0.05866
*CCL8*	C-C motif chemokine ligand 8	*APLNR*	apelin receptor	-0.02258
*TINAGL1*	tubulointerstitial nephritis antigen like 1	*IGF2*	insulin-like growth factor 2	-0.22219
*APOBEC3G*	apolipoprotein B mRNA editing enzyme catalytic subunit 3G	*ACVRL1*	activin A receptor-like type 1	-0.00508
*APOBEC3G*	apolipoprotein B mRNA editing enzyme catalytic subunit 3G	*ANGPTL2*	angiopoietin-like 2	-0.13337
*APOBEC3G*	apolipoprotein B mRNA editing enzyme catalytic subunit 3G	*S1PR1*	sphingosine-1-phosphate receptor 1	-0.12991
*TLR2*	toll-like receptor 2	*DLL4*	delta-like canonical Notch ligand 4	-0.00407
*PAEP*	progestagen associated endometrial protein	*PTK2B*	protein tyrosine kinase 2 beta	0.059944
*PAEP*	progestagen associated endometrial protein	*TYMP*	thymidine phosphorylase	0.125313
*FABP3*	fatty acid-binding protein 3	*MDK*	Midkine	0.212626
*IRF1*	interferon regulatory factor 1	*MET*	MET proto-oncogene, receptor tyrosine kinase	-0.32083
*APOBEC3F*	apolipoprotein B mRNA editing enzyme catalytic subunit 3F	*EDNRA*	endothelin receptor type A	-0.14079
*LYZ*	lysozyme	*NEO1*	neogenin 1	-0.04497
*APOBEC3H*	apolipoprotein B mRNA editing enzyme catalytic subunit 3H	*CXCR6*	C-X-C motif chemokine receptor 6	0.038609
*MARCO*	macrophage receptor with collagenous structure	*PLAUR*	plasminogen activator, urokinase receptor	-0.18686
*IRF7*	interferon regulatory factor 7	*RAC3*	Rac family small GTPase 3	-0.10045
*PLSCR1*	phospholipid scramblase 1	*RAC3*	Rac family small GTPase 3	-0.14566
*CXCR6*	C-X-C motif chemokine receptor 6	*IL24*	interleukin 24	-0.08622
*LTBP1*	latent-transforming growth factor beta-binding protein 1	*LHB*	luteinizing hormone subunit beta	-0.09531
*CCRL2*	C-C motif chemokine receptor-like 2	*CTLA4*	cytotoxic T-lymphocyte associated protein 4	0.092781
*CLCF1*	corticotrophin like cytokine factor 1	*LHB*	luteinizing hormone subunit beta	-0.2615
*IL1RN*	interleukin 1 receptor antagonist	*LCP2*	lymphocyte cytosolic protein 2	0.370091

**Table 2:** Summary of the results of univariate and multivariate analyses of the risk factors for the OS of patients with melanoma in the TCGA-SKCM cohort, the TCGA-UVM cohort, and the GEO cohort.

Datasets	Variables	Univariate analysis		Multivariate analysis	
		HR (95% CI)	P‑value	HR (95% CI)	P‑value
TCGA-SKCM (Training dataset)	Age	1.023(1.007−1.039)	0.005	1.012(0.997−1.028)	0.129
	Gender	1.297(0.770−2.185)	0.328	1.115(0.645−1.926)	0.697
	Stage	1.538(1.205−1.962)	<0.001	1.210(0.557−2.625)	0.630
	T stage	1.828(1.431−2.337)	<0.001	1.428(0.885−2.305)	0.145
	N stage	1.546(1.212−1.973)	<0.001	0.970(0.526−1.791)	0.923
	Clark level	1.506(1.128−2.011)	0.006	0.819(0.565−1.188)	0.293
	Risk-score	3.831(2.726−5.383)	<0.001	3.453(2.392−4.985)	<0.001
GSE65904 (Validation dataset)	Age	0.997(0.982−1.012)	0.713	1.000(0.985−1.015)	0.986
	Gender	1.302(0.832−2.039)	0.249	1.316(0.838−2.065)	0.233
	Stage	1.405(1.167−1.692)	<0.001	1.457(1.211−1.754)	<0.001
	Risk-score	1.723(1.226−2.423)	0.002	1.902(1.319−2.744)	<0.001
TCGA-UVM (Validation dataset)	Age	1.046(1.008–1.085)	0.019	1.051(1.012–1.092)	0.010
	Gender	1.542(0.651–3.652)	0.325	1.376(0.563–3.366)	0.484
	Stage	1.311(0.839–2.048)	0.234	1.392(0.862–2.246)	0.176
	T stage	1.941(0.712–5.294)	0.195	1.506(0.529–4.291)	0.443
	Risk-score	5.213(1.686–16.120)	0.004	7.645(2.054–28.452)	0.002

Abbreviations: HR, hazard ratio; CI, confidence interval

### Signature validation in the GEO dataset

Using the risk score cut-off, we stratified the patients in the GEO validation cohort into high- and low-risk groups. Consistent with the findings previously obtained in the TCGA dataset, a significant difference of OS was found between the two groups (P = 2.1×10^−3^, HR = 1.988 (1.309 to 3.020)) (see in Fig 2B). Similarly, we found clinical factors, including age and gender, didn't exhibit a predictive value in both the univariate and multivariate Cox analysis (P >0.01) (see in Table 2 and Fig 3B). Notably, the IRGPs signature remained an independent predictive value of OS (see in Table 2 and Fig 3B) in both the univariate and multivariate Cox analysis in the validation dataset (P<0.01).

### Signature validation in the TCGA-UVM dataset

Similar to the GEO dataset, the TCGA-UVM dataset (n = 75) was stratified into high- and low-risk groups according to the cut-off value. Notably, after grouping, most patients were in the high-risk group; there were only eight patients in the low-risk group in this dataset. The trend in the results of the survival analysis in TCGA-UVM was as we expected (patients in the high-risk group may exhibit a poorer prognosis), but the result was not statistically significant (P = 0.117, HR = 4.263 (1.407 to 12.91)) (see in Fig 2C). However, the IRGPs signature remained an independent predictive value of OS (see in Table 2 and Fig 3C) in both the univariate and multivariate Cox analysis in this validation dataset (P<0.01).

### Immune cell infiltration between different risk groups

**Fig 5:**
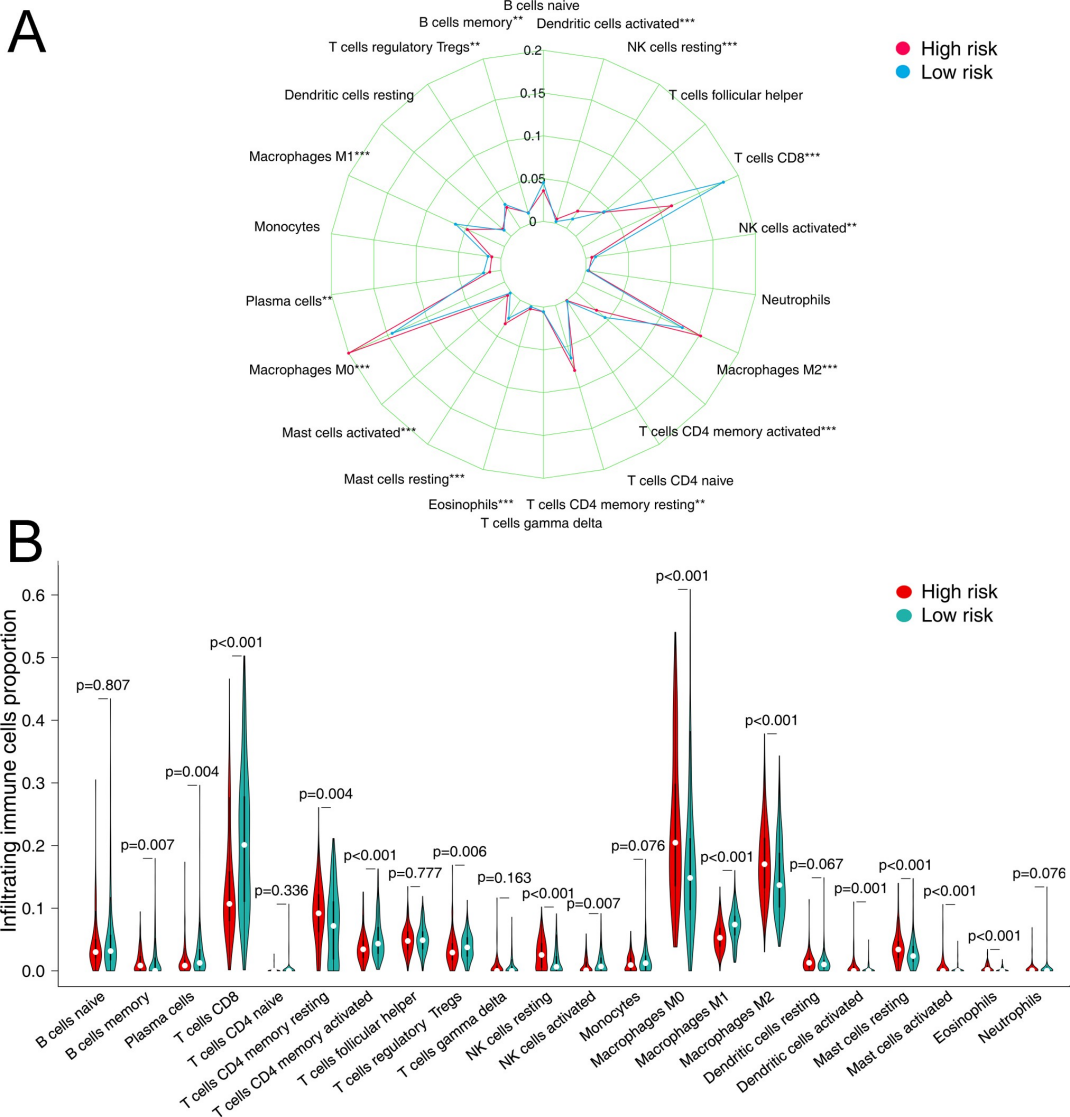
The relative fraction of infiltrated immune cells in different risk groups in the TCGA dataset. (A) Radar plot of the difference in the abundance of 22 immune cells in tumor tissue in the two risk groups. (B) Violin plot of differences in various immune cell abundances between the high- and low-risk groups. (*FDR adjusted P-value<0.05, **FDR adjusted P-value <0.01, ***FDR adjusted P-value<0.001).

CIBERSORTx was used to estimate the fractions of 22 infiltrated immune cells using the RNA-sequence data. We used a threshold of P < 0.05 to rule out unreliable results. Among the 378 tumor samples in TCGA, only 374 tumor samples were eligible for further analysis. The relative abundance of parts of the 22 infiltrated immune cells exhibited significant differences between the high- and low-risk groups. When compared to other immune cells in tumor tissues, CD8 T cells, resting CD4 memory T cells, macrophages M0, and macrophages M2 are the four immune cells with the highest abundance (relative fraction >0.05 in both risk groups) (see in Fig 5A and 5B). Compared to the low-risk group, the proportion of M0 Macrophages, M2 Macrophages, resting CD4 memory T cells, resting NK cells, Mast cells resting, Eosinophils, and activated Mast cells exhibited higher fraction in the high-risk group (FDR adjusted P value<0.01). Conversely, CD8 T cells, activated DCs, regulatory T cells (Tregs), and activated CD4 memory T cells presented a significantly lower fraction in the high-risk group (FDR adjusted P value <0.01) (see in Fig 5A and 5B).

### Functional characteristics of the IRGPs signature

**Fig 6:**
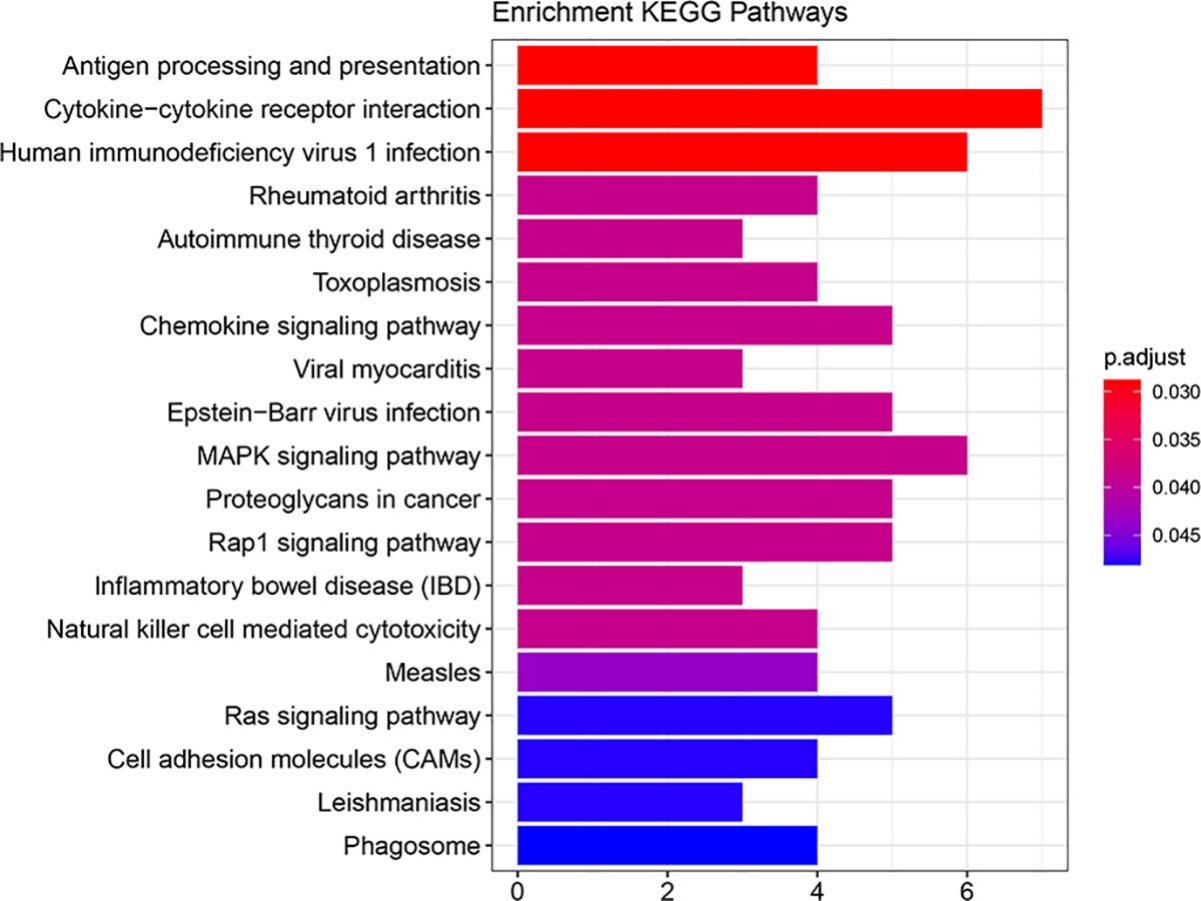
The KEGG pathway enrichment analysis of 52 immune-related genes. Significantly, there are seven genes enriched in the pathway of cytokine-cytokine receptor interactions. KEGG, Kyoto Encyclopedia of Genes and Genomes.

**Fig 7:**
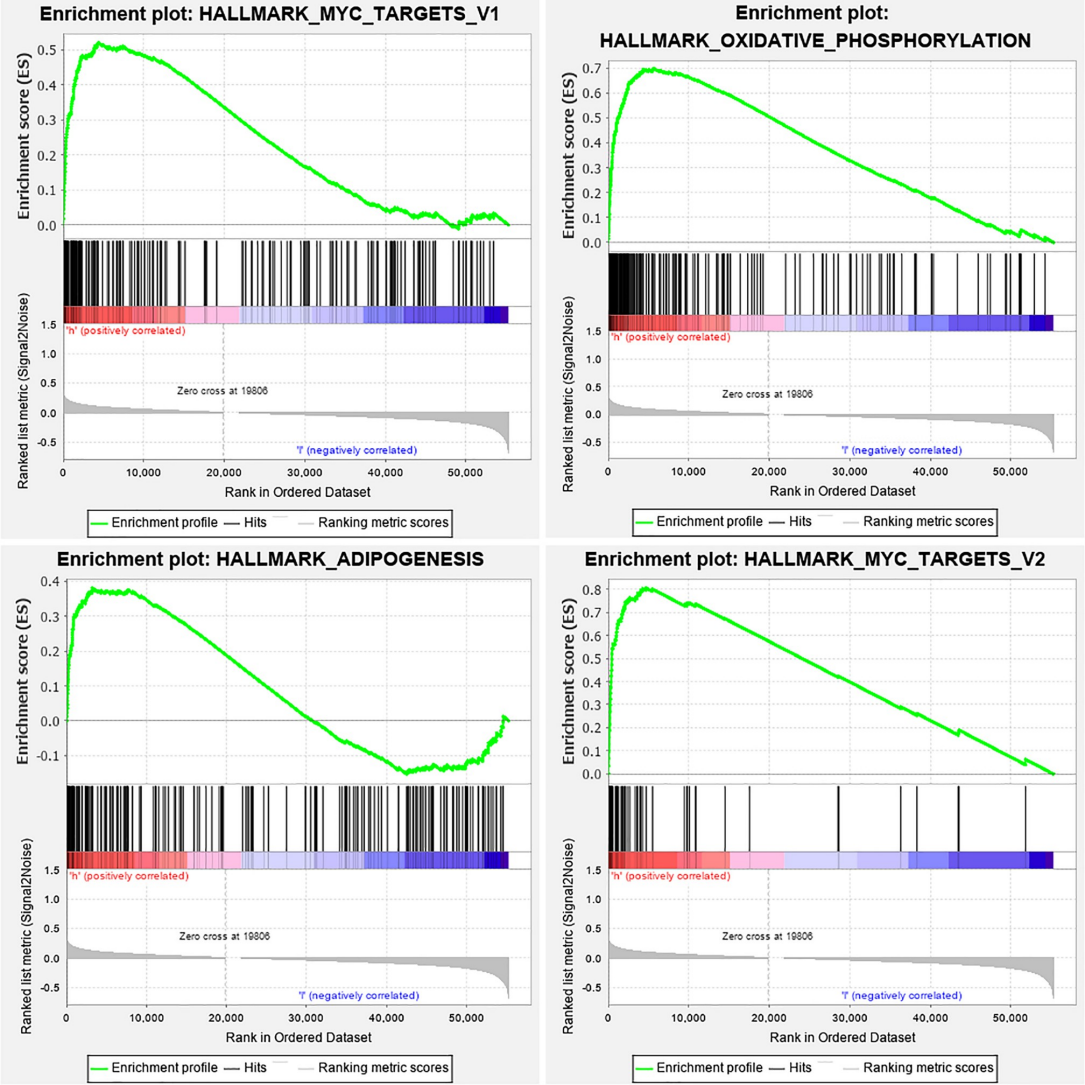
GSEA enrichment analysis of the TCGA cohort with hallmark gene sets. According to the GSEA results, there were four significant gene sets enrichments in the high-risk group (P < 0.05, FDR < 0.25). GSEA, Gene set enrichment analysis.

Since the IRGPs signature obtained was found to be highly correlated with melanoma development and prognosis, then we attempted to explore their functional implication and intrinsic association through subsequent enrichment analysis. Results of KEGG functional enrichment analysis revealed that 52 related genes of the signature were enriched significantly in 19 KEGG pathways (P<0.05). Among them, the pathway of Cytokine-cytokine receptor interaction was most significantly enriched (see in Fig 6 and S2 Table). We classified patients into high- and low-risk groups based on IRGPs risk scores cut-off value and used this risk classification as a phenotype for GSEA enrichment analysis of the TCGA cohort. As a result, we found the enrichment of four cancer hallmark gene sets in the high-risk group, including "OXIDATIVE_PHOSPHORYLATION", "ADIPOGENESIS", "MYC_TARGETS_V1", and "MYC_TARGETS_V2" (see in Fig 7), which suggests a crucial role in melanoma progression and prognosis of these significantly enriched gene sets.

## Discussion

The annual incidence of melanoma has been increasing, which may be related to an increased detection rate of the disease. In the United States, according to the latest report, about 95,710 newly discovered melanoma in situ cases will be diagnosed in 2020, and about 7,230 of them will likely die from the disease [3]. The 5-year survival rate for cutaneous melanoma is relatively optimistic, at approximately 92% [3]. However, the mortality rate is significantly higher if the melanoma metastasizes subcutaneously to regional lymph nodes or distant organs [21]. Recently, many studies have proposed different genes markers or signatures to predict the metastasis or survival of malignant melanoma. Yang et al. constructed a robust prognostic signature of six lncRNAs for melanoma patients. They suggested that the prognosis may be affected by these lncRNAs by regulating immune and inflammation-related pathways, MAPK pathways, and others [22]. Another four-DNA methylation signature significantly correlated with prognosis was proposed by Guo et al. The marker was also found to be substantially associated with immunotherapy-associated features of immune checkpoint blockade and was considered a potential indicator of immunotherapy responsiveness [23]. Sheng et al. tried to predict melanoma metastasis by constructing an immune-related risk model and successfully validated its efficacy in different datasets [1]. However, the clinical applicability of these biomarkers remains limited due to tumor heterogeneity and sequencing technical problems. In particular, the issue of standardization of the data from different sequencing platforms is also a challenge in clinical applications. Therefore, in our study, to eliminate the influence of different platforms and inter-individual standardization on the results, we introduced the concept of immune gene pairs. And by assigning the size of a specific pair of immune gene expression values, we obtained a new predictive model that is more suitable for individual studies and clinical application. As described by Li et al., there is no need for data normalization or to consider technical bias across platforms as it only performs pairwise comparisons of the expression values of the target genes within a single sample [17]. Several previous studies have confirmed the availability and accuracy of this immune gene pairs method in predicting OS in different types of cancer, including hepatocellular carcinoma and serous ovarian carcinoma [14–17]. In this study, we successfully constructed a robust prognostic signature consisting of 33 IRGPs using the TCGA-SKCM dataset. And its predictive efficacy has been validated in two external datasets (GSE65904 and TCGA-UVM). Although the prognosis of patients in the high-risk group was also relatively poorer, the result of the survival analysis in the TCGA-UVM dataset was not significant (P >0.05). To explain this, we believe that this is closely related to the disproportionately low number of patients in the low-risk group (n = 8). For the signature was confirmed in subsequent univariate and multivariate regression analyses as an independent prognostic indicator significantly superior to clinical factors such as age or staging (Fig 3C). Abundant evidence suggests the close relationship between the immune system and tumor development or prognosis. Immunotherapy plays a critical role in prolonging the survival time of advanced melanoma. Recently, the discovery and application of immune checkpoint inhibitors had been a boon to patients with melanoma and other malignancies [2]. Immunological findings have given us new hopes for a complete cure for the tumor. The main reason why tumor cells are difficult to destroy is their ability to escape from the immune system through different mechanisms, and the strength of the body's anti-tumor immune response directly affects the prognosis and outcome of tumor patients [2, 24, 25]. Therefore, it is logical and accurate to predict tumor development, prognosis, and outcome by selecting immune-related genes.

In our study, A robust signature of 33 IRGPs consist of 52 IRGs was identified to predict OS for melanoma patients. Nearly half of the genes that make up this marker are related to cytokines or chemokines. Among the IRGs filtered, the *CTLA-4* promotes immune escape of tumor cells by competitively binding to *CD80* and *CD86*, blocking T cell activation and anti-tumor immune response of the body [24, 26]. The chemokine receptor *CXCR6* is selectively expressed on T cells, plasma cells, and NKs. In melanoma, *CXCR6* is overexpressed in primary and metastatic melanoma, and *CXCR6* positive cells are identified as cancer stem cells and can self-renew but also generate other tumor cells [27]. *IL-24* belongs to the *IL-10* family of cytokines that inhibits tumor cell growth and promotes tumor cell apoptosis by inhibiting angiogenesis, activating growth inhibition and DNA damage genes, and other signaling pathways, without harming healthy cells [28]. While *MET* receptors, also known as *HGF* receptors, are mainly expressed in epithelial cells of many tissues, including the skin, oncogenic effects of the *HGF/MET* signaling pathway has long been observed in melanoma and other malignancies [29]. Thus, beneficial anti-tumor effects may be produced by inhibiting the *MET* signaling pathway. Related targeted therapies have been studied extensively and have yielded impressive results. The more widely used ones, such as Tivantinib, are *MET* pathway inhibitors that bind to dephosphorylated *MET* kinases and inhibit the activation and transduction of *MET* signaling pathways. Its safety and efficacy have been staunchly demonstrated in clinical trials in advanced melanoma [30].

Tumor-infiltrating lymphocytes (TILs) are those leukocytes (NK cells, myeloid-derived suppressor cells (MDSCs), B cells, T cells, macrophages, DCs, and others) that leave the bloodstream and enter the tumor tissue. When a large amount of tumor-infiltrating lymphocytes are present, it indicates that the organism has initiated an immune response against the tumor. Researchers have conducted relevant studies in many of these cancers, quantifying these tumor-infiltrating cells and correlating their abundance with tumor features and outcomes [31]. Previous studies have provided substantial evidence to support a favorable prognosis and outcome for malignant melanoma with abundant infiltration of TILs [32, 33]. In the current study, by using the CIBERSORTx platform, we have estimated the relative fractions of 22 TILs in tumor tissues from the TCGA cohort using the CIBERSORTx platform. Using the IRPGs risk score cut-off, we divided the CIBERSORTx result into two different risk groups and then verified the correlations of immune cells fraction with the IRPGs risk factor. Significant differences in the relative fraction of infiltrated immune cells in tumor tissue were observed between the two different risk groups. The highest concentration of lymphocytes in tumor tissue is found in macrophages M0, macrophages M2 and CD8 T cells compared to other immune cells (relative portion>0.1 in both risk groups). Compared with the high-risk group, Eosinophils, M0 Macrophages, M2 Macrophages, and activated Mast cells presented a lower fraction in the low-risk group. The primary function of macrophages is phagocytosis and digestion of cellular debris and pathogens, and activation of other immune cells. However, tumor-associated macrophages (TAM) not only prevent T cells from attacking tumor cells but also secrete growth factors that nourish tumor cells and promote tumor angiogenesis, leading to tumor cell expansion and metastasis [34–36]. M0 macrophage is an inactivated macrophage that without any inflammatory or tumor-associated function. However, due to the state of activation and roles in the tumor microenvironment, M0 macrophages can be transformed into classically activated M1 macrophages and alternatively activated M2 macrophages. And the difference is M1 macrophages have mainly anti-tumor effects and can differentiate tumor cells from healthy cells, recognize and then kill tumor cells by mediating cytotoxic effects. The role of M2 macrophages is, on the contrary, to promote tumor growth and metastasis. It can secrete cytokines such as epidermal factors to stimulate the proliferation of tumor cells, and also facilitate tumor angiogenesis and migration of tumor cells multiple regulatory pathways [34]. In our study, we found more M0 macrophages and M2 macrophage infiltrates in the high-risk group, indicating a more suitable microenvironment for tumor growth and metastasis, resulting in a poor prognosis for patients. Both eosinophils and mast cells belong to inflammatory cells. These inflammatory cells are sought to contribute to barriers to anti-tumor immunity [37]. Cause the inflammatory environment in the tumor tissue is believed to promote the development and progression of the tumor according to previous studies [38]. Conversely, in the high-risk group of our research, activated memory CD4+ T cells, CD8+ T cells, activated DCs, and Tregs presented a significantly lower fraction. Significant infiltration of CD8+ T cells among TILs suggested a more robust anti-tumor immune response with a better prognosis in the low-risk group.

The CIBERSORTx software imputes cell fractions for 22 immune cell types based on a signature matrix file consisting of 547 genes. Interestingly, we found seven genes in the IRGPs signature (*APOBEC3G*, *CCL13*, *CCL8*, *CTLA4*, *CXCR6*, *MARCO*, *TLR2*) that are also in the CIBERSORTx reference gene list. All six genes belong to antimicrobials except *CTLA4*, which is a gene related to the T-cell receptor signaling pathway [39]. This suggests that antimicrobials are significantly correlated with both the composition of immune cells and tumor prognosis. It also explains why the prognostic signature we obtained was significantly correlated with the fraction of multiple immune cells. The primary purpose of the signature is to synthesize and distill these factors related to overall survival and to obtain, when possible, a brief and comprehensive evaluation indicator.

Consistent with previous studies, GSEA enrichment analysis of the TCGA cohort revealed that four cancer hallmark gene sets, including "ADIPOGENESIS" were significantly enriched in the high-risk group, suggesting a crucial role in melanoma progression and metastasis of these gene sets [14, 40, 41].

There are several limitations to the present study. Firstly, this study was not a prospective study. But we try to verify the accuracy of our results by recruiting the other two external datasets for validation. Second, Gene expression testing is expensive and requires tumor tissue to be obtained in advance, so it is challenging to apply it routinely in clinical practice. Furthermore, the signature we established in this study consisted of 52 immune-related genes that needed to be tested simultaneously, which resulted in the inability to validate them in some other datasets. The signature should also be optimized with more rigorous algorithms and validated with more datasets from different platforms.

## Conclusion

We constructed a robust prognosis-related signature for melanoma using a novel immune gene pair approach and successfully validated its efficacy in other external datasets. This approach is more suitable for scientific research and clinical guidance as it only compares the values of gene expression profiles within a single sample and does not require a standardization of the data or consideration of technical bias across platforms.

## Supporting information

S1 Fig Forest plots of univariate and multivariate Cox regression analyses in different sub-cohorts in the TCGA-SKCM dataset. (A) *BRAF*-mutated patients (n = 156). (B) *BRAF*-wild-type patients (n = 222). (C) *NRAS*-mutated patients (n = 87). (D) *NRAS*-wild-type patients(n = 291). HR, hazard ratio.
(TIF)

S1 Table Patient clinical and pathologic characteristics for the training and validation datasets. (DOCX)

S2 Table KEGG pathway analysis of genes from the IRGPs signature. (DOCX)

S1 Raw images (PDF)
