# Supplementary figures and images for: Predicting the clinical outcome of melanoma using an immune-related gene pairs signature
Source: PLoS One. 2020 Oct 8;15(10):e0240331. doi: 10.1371/journal.pone.0240331 (PMC7544036; doi:10.1371/journal.pone.0240331)

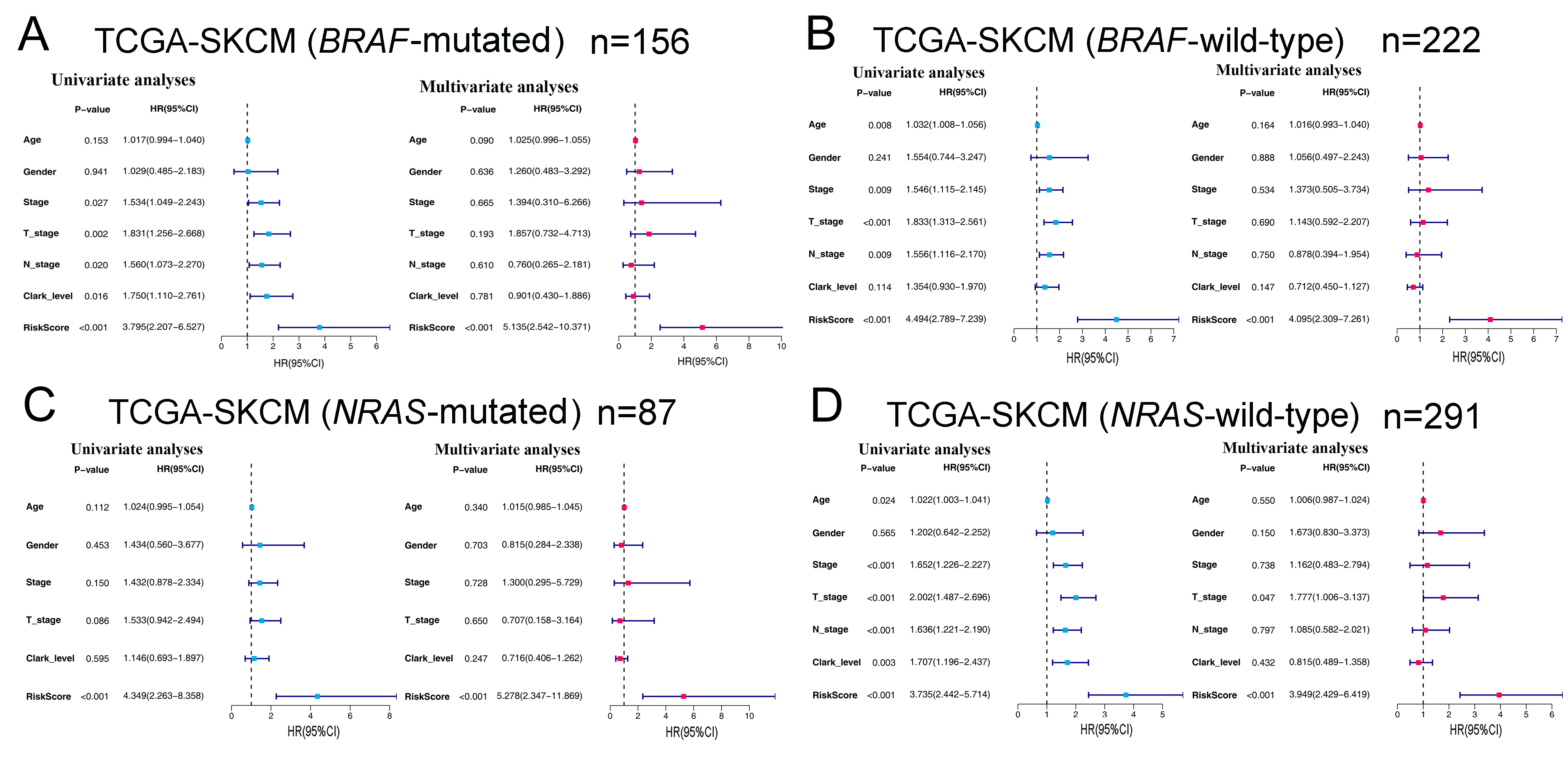

Supplement: S1 Fig — (A) BRAF-mutated patients (n = 156). (B) BRAF-wild-type patients (n = 222). (C) NRAS-mutated patients (n = 87). (D) NRAS-wild-type patients(n = 291). HR, hazard ratio. (TIF) [file pone.0240331.s001.tif]
