# Supplementary material for: Predicting the clinical outcome of melanoma using an immune-related gene pairs signature
Source: PLoS One. 2020 Oct 8;15(10):e0240331. doi: 10.1371/journal.pone.0240331 (PMC7544036; doi:10.1371/journal.pone.0240331)
Supplement: S1 Table — (DOCX) [file pone.0240331.s002.docx]

**Table S1 Patient clinical and pathologic characteristics for the training and validation cohorts.**

|  | | Training cohort | Validation cohorts | |
| --- | --- | --- | --- | --- |
|  |  | TCGA-SKCM (n=378) | GSE65904 (n=186) | TCGA-UVM (n=75) |
| Age (years, mean) | | 56.4 (15-87) | 62.5 (22-91) | 61.9 (22-86) |
| Gender | Male | 246 (65.1%) | 100 (53.8%) | 43 (57.3%) |
|  | Female | 132 (34.9%) | 86 (46.2%) | 32 (42.7%) |
| Stage | I-II | 125 (33.1%) | 130 (69.9%) | 35 (46.7) |
|  | III-IV | 167 (44.2%) | 49 (26.3%) | 39 (52%) |
|  | Unknown | 86 (22.8%) | 7 (3.8%) | 1 (1.3%) |
| T stage | I-II | 112 (29.6%) |  | 12 (16%) |
|  | III-IV | 173 (45.8%) |  | 62 (82.7%) |
|  | Unknown | 93 (24.6%) |  | 1 (1.3%) |
| N stage | NO | 182 (48.1%) |  |  |
|  | N1-2 | 108 (28.6%) |  |  |
|  | Unknown | 88 (23.3%) |  |  |
| Clark level | C1-3 | 88 (23.3%) |  |  |
|  | C4-5 | 180 (47.6%) |  |  |
|  | Unknown | 110 (29.1%) |  |  |
| Radiation therapy | Yes | 73 (19.3%) |  |  |
|  | No | 285 (75.4%) |  |  |
|  | Unknown | 20 (5.3%) |  |  |
| BRAF mutation | Wild-type | 222 (58.7%) |  |  |
|  | Mutated | 156 (41.3%) |  |  |
| NRAS mutation | Wild-type | 291 (77.0%) |  |  |
|  | Mutated | 87 (23.0%) |  |  |
