# Supplementary material for: Predicting the clinical outcome of melanoma using an immune-related gene pairs signature
Source: PLoS One. 2020 Oct 8;15(10):e0240331. doi: 10.1371/journal.pone.0240331 (PMC7544036; doi:10.1371/journal.pone.0240331)
Supplement: S2 Table — (DOCX) [file pone.0240331.s003.docx]

**Table S2. KEGG pathway analysis of genes from the IRGPs signature**

| ID | Description | GeneRatio | BgRatio | p-value | p.adjust | q-value | Gene symbol | Count |
| --- | --- | --- | --- | --- | --- | --- | --- | --- |
| hsa04612 | Antigen processing and presentation | 4/42 | 78/8037 | 0.000695 | 0.028787 | 0.021682 | HLA-DQB1/HSPA2/HLA-DQA2/PSME1 | 4 |
| hsa04060 | Cytokine-cytokine receptor interaction | 7/42 | 294/8037 | 0.000732 | 0.028787 | 0.021682 | ACVRL1/CCL8/CLCF1/CXCR6/IL1RN/IL24/CCL13 | 7 |
| hsa05170 | Human immunodeficiency virus 1 infection | 6/42 | 212/8037 | 0.000745 | 0.028787 | 0.021682 | TLR2/APOBEC3G/APOBEC3H/APOBEC3F/RAC3/PTK2B | 6 |
| hsa05323 | Rheumatoid arthritis | 4/42 | 93/8037 | 0.001344 | 0.038721 | 0.029164 | HLA-DQB1/TLR2/CTLA4/HLA-DQA2 | 4 |
| hsa05320 | Autoimmune thyroid disease | 3/42 | 53/8037 | 0.002593 | 0.038721 | 0.029164 | HLA-DQB1/CTLA4/HLA-DQA2 | 3 |
| hsa05145 | Toxoplasmosis | 4/42 | 112/8037 | 0.002661 | 0.038721 | 0.029164 | HLA-DQB1/TLR2/HSPA2/HLA-DQA2 | 4 |
| hsa04062 | Chemokine signaling pathway | 5/42 | 189/8037 | 0.002864 | 0.038721 | 0.029164 | CCL8/RAC3/PTK2B/CXCR6/CCL13 | 5 |
| hsa05416 | Viral myocarditis | 3/42 | 60/8037 | 0.003693 | 0.038721 | 0.029164 | HLA-DQB1/RAC3/HLA-DQA2 | 3 |
| hsa05169 | Epstein-Barr virus infection | 5/42 | 201/8037 | 0.003733 | 0.038721 | 0.029164 | HLA-DQB1/TLR2/OAS1/HLA-DQA2/IRF7 | 5 |
| hsa04010 | MAPK signaling pathway | 6/42 | 294/8037 | 0.003935 | 0.038721 | 0.029164 | HSPA2/RAC3/FGF1/MET/IGF2/KDR | 6 |
| hsa05205 | Proteoglycans in cancer | 5/42 | 205/8037 | 0.004061 | 0.038721 | 0.029164 | TLR2/MET/IGF2/KDR/PLAUR | 5 |
| hsa04015 | Rap1 signaling pathway | 5/42 | 210/8037 | 0.004499 | 0.038721 | 0.029164 | RAC3/FGF1/MET/KDR/LCP2 | 5 |
| hsa05321 | Inflammatory bowel disease (IBD) | 3/42 | 65/8037 | 0.004629 | 0.038721 | 0.029164 | HLA-DQB1/TLR2/HLA-DQA2 | 3 |
| hsa04650 | Natural killer cell mediated cytotoxicity | 4/42 | 131/8037 | 0.004673 | 0.038721 | 0.029164 | MICB/RAC3/PTK2B/LCP2 | 4 |
| hsa05162 | Measles | 4/42 | 138/8037 | 0.005619 | 0.043452 | 0.032727 | TLR2/OAS1/HSPA2/IRF7 | 4 |
| hsa04014 | Ras signaling pathway | 5/42 | 232/8037 | 0.006836 | 0.047841 | 0.036033 | RAC3/FGF1/MET/IGF2/KDR | 5 |
| hsa04514 | Cell adhesion molecules (CAMs) | 4/42 | 148/8037 | 0.007181 | 0.047841 | 0.036033 | HLA-DQB1/NEO1/CTLA4HLA-DQA2 | 4 |
| hsa05140 | Leishmaniasis | 3/42 | 77/8037 | 0.007424 | 0.047841 | 0.036033 | HLA-DQB1/TLR2/HLA-DQA2 | 3 |
| hsa04145 | Phagosome | 4/42 | 152/8037 | 0.007878 | 0.0481 | 0.036228 | HLA-DQB1/TLR2/MARCO/HLA-DQA2 | 4 |

**Abbreviations:**KEGG, Kyoto Encyclopedia of Genes and Genomes; IRGPs, immune-related gene pairs.
