# Supplementary material for: Predicting the clinical outcome of melanoma using an immune-related gene pairs signature
Source: PLoS One. 2020 Oct 8;15(10):e0240331. doi: 10.1371/journal.pone.0240331 (PMC7544036; doi:10.1371/journal.pone.0240331)
Supplement: S1 Raw images — (PDF) [file pone.0240331.s004.pdf]

Fig 1

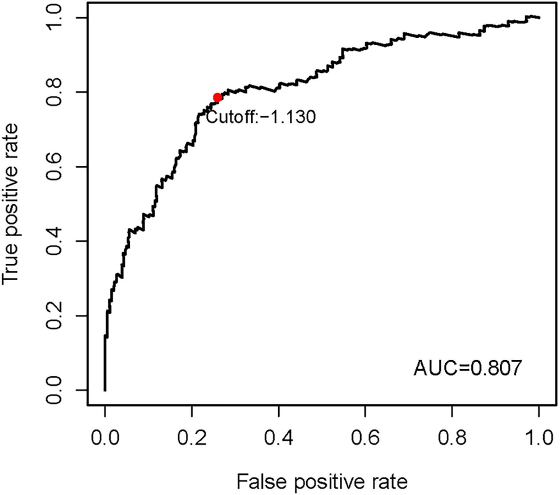

True positive rate
False positive rate
0.0
0.2
0.4
0.6
0.8
1.0
Cutoff:−1.130
AUC=0.807

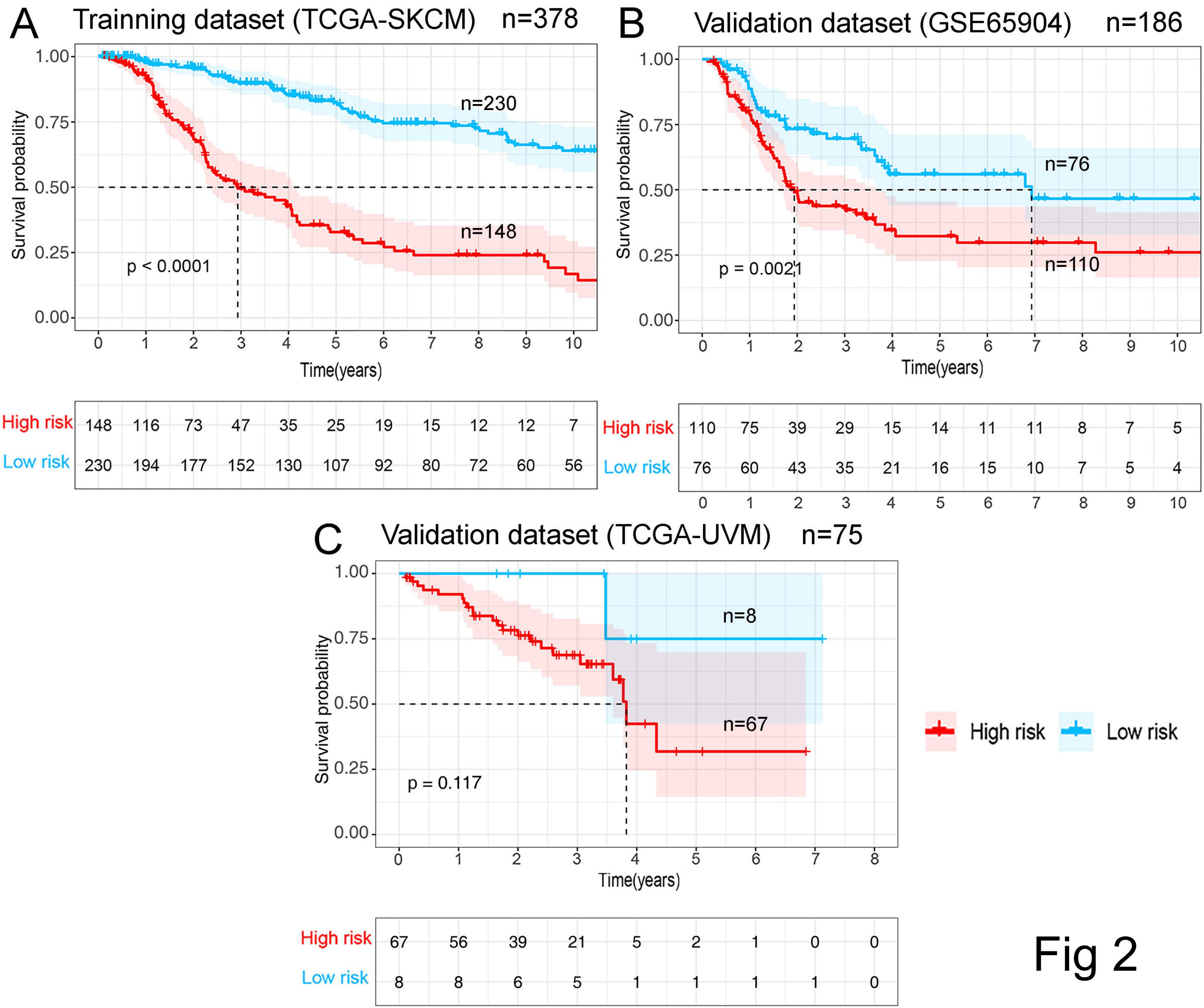
A
Trainning dataset (TCGA-SKCM) n=378
Survival probability
1.00
0.75
0.50
0.25
0.00
n=230
n=148
p < 0.0001
Time(years)
0 1 2 3 4 5 6 7 8 9 10
High risk 148 116 73 47 35 25 19 15 12 12 7
Low risk 230 194 177 152 130 107 92 80 72 60 56
B
Validation dataset (GSE65904) n=186
Survival probability
1.00
0.75
0.50
0.25
0.00
n=76
n=110
p = 0.0021
Time(years)
0 1 2 3 4 5 6 7 8 9 10
High risk 110 75 39 29 15 14 11 11 8 7 5
Low risk 76 60 43 35 21 16 15 10 7 5 4
0 1 2 3 4 5 6 7 8 9 10
C
Validation dataset (TCGA-UVM) n=75
Survival probability
1.00
0.75
0.50
0.25
0.00
n=8
n=67
p = 0.117
Time(years)
0 1 2 3 4 5 6 7 8
High risk 67 56 39 21 5 2 1 0 0
Low risk 8 8 6 5 1 1 1 1 0
High risk
Low risk

Fig 2

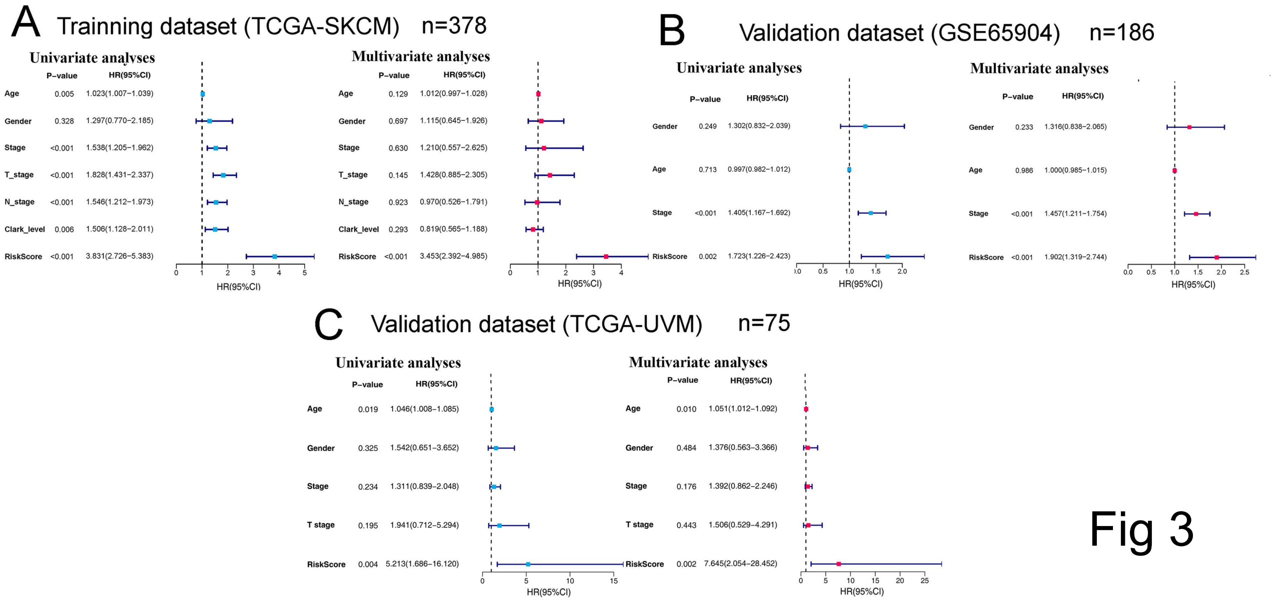
A
Trainning dataset (TCGA-SKCM) n=378
Univariate analyses
P-value HR(95%CI)
Age 0.005 1.023(1.007-1.039)
Gender 0.328 1.297(0.770-2.185)
Stage <0.001 1.538(1.205-1.962)
T_stage <0.001 1.828(1.431-2.337)
N_stage <0.001 1.546(1.212-1.973)
Clark_level 0.006 1.506(1.128-2.011)
RiskScore <0.001 3.831(2.726-5.383)
HR(95%CI)
Multivariate analyses
P-value HR(95%CI)
Age 0.129 1.012(0.997-1.028)
Gender 0.697 1.115(0.645-1.926)
Stage 0.630 1.210(0.557-2.625)
T_stage 0.145 1.428(0.885-2.305)
N_stage 0.923 0.970(0.526-1.791)
Clark_level 0.293 0.819(0.565-1.188)
RiskScore <0.001 3.453(2.392-4.985)
HR(95%CI)
B
Validation dataset (GSE65904) n=186
Univariate analyses
P-value HR(95%CI)
Gender 0.249 1.302(0.832-2.039)
Age 0.713 0.997(0.982-1.012)
Stage <0.001 1.405(1.167-1.692)
RiskScore 0.002 1.723(1.226-2.423)
HR(95%CI)
Multivariate analyses
P-value HR(95%CI)
Gender 0.233 1.316(0.838-2.065)
Age 0.986 1.000(0.985-1.015)
Stage <0.001 1.457(1.211-1.754)
RiskScore <0.001 1.902(1.319-2.744)
HR(95%CI)
C
Validation dataset (TCGA-UVM) n=75
Univariate analyses
P-value HR(95%CI)
Age 0.019 1.046(1.008-1.085)
Gender 0.325 1.542(0.651-3.652)
Stage 0.234 1.311(0.839-2.048)
T stage 0.195 1.941(0.712-5.294)
RiskScore 0.004 5.213(1.686-16.120)
HR(95%CI)
Multivariate analyses
P-value HR(95%CI)
Age 0.010 1.051(1.012-1.092)
Gender 0.484 1.376(0.563-3.366)
Stage 0.176 1.392(0.862-2.246)
T stage 0.443 1.506(0.529-4.291)
RiskScore 0.002 7.645(2.054-28.452)
HR(95%CI)

Fig 3

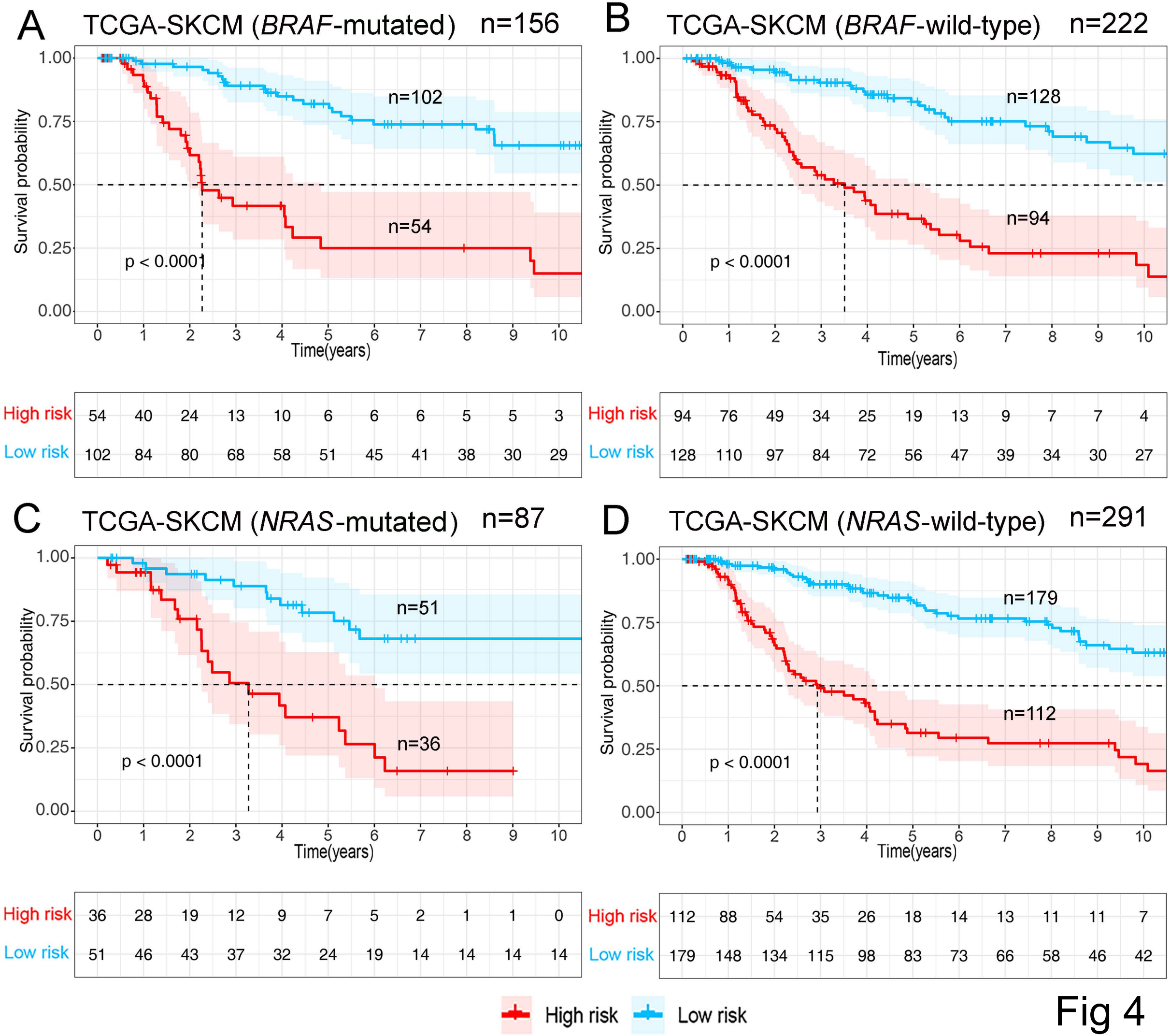
A
TCGA-SKCM (BRAF-mutated) n=156
Survival probability
Time(years)
n=102
n=54
p < 0.0001
High risk 54 40 24 13 10 6 6 6 5 5 3
Low risk 102 84 80 68 58 51 45 41 38 30 29
B
TCGA-SKCM (BRAF-wild-type) n=222
Survival probability
Time(years)
n=128
n=94
p < 0.0001
High risk 94 76 49 34 25 19 13 9 7 7 4
Low risk 128 110 97 84 72 56 47 39 34 30 27
C
TCGA-SKCM (NRAS-mutated) n=87
Survival probability
Time(years)
n=51
n=36
p < 0.0001
High risk 36 28 19 12 9 7 5 2 1 1 0
Low risk 51 46 43 37 32 24 19 14 14 14 14
D
TCGA-SKCM (NRAS-wild-type) n=291
Survival probability
Time(years)
n=179
n=112
p < 0.0001
High risk 112 88 54 35 26 18 14 13 11 11 7
Low risk 179 148 134 115 98 83 73 66 58 46 42
High risk
Low risk

Fig 4

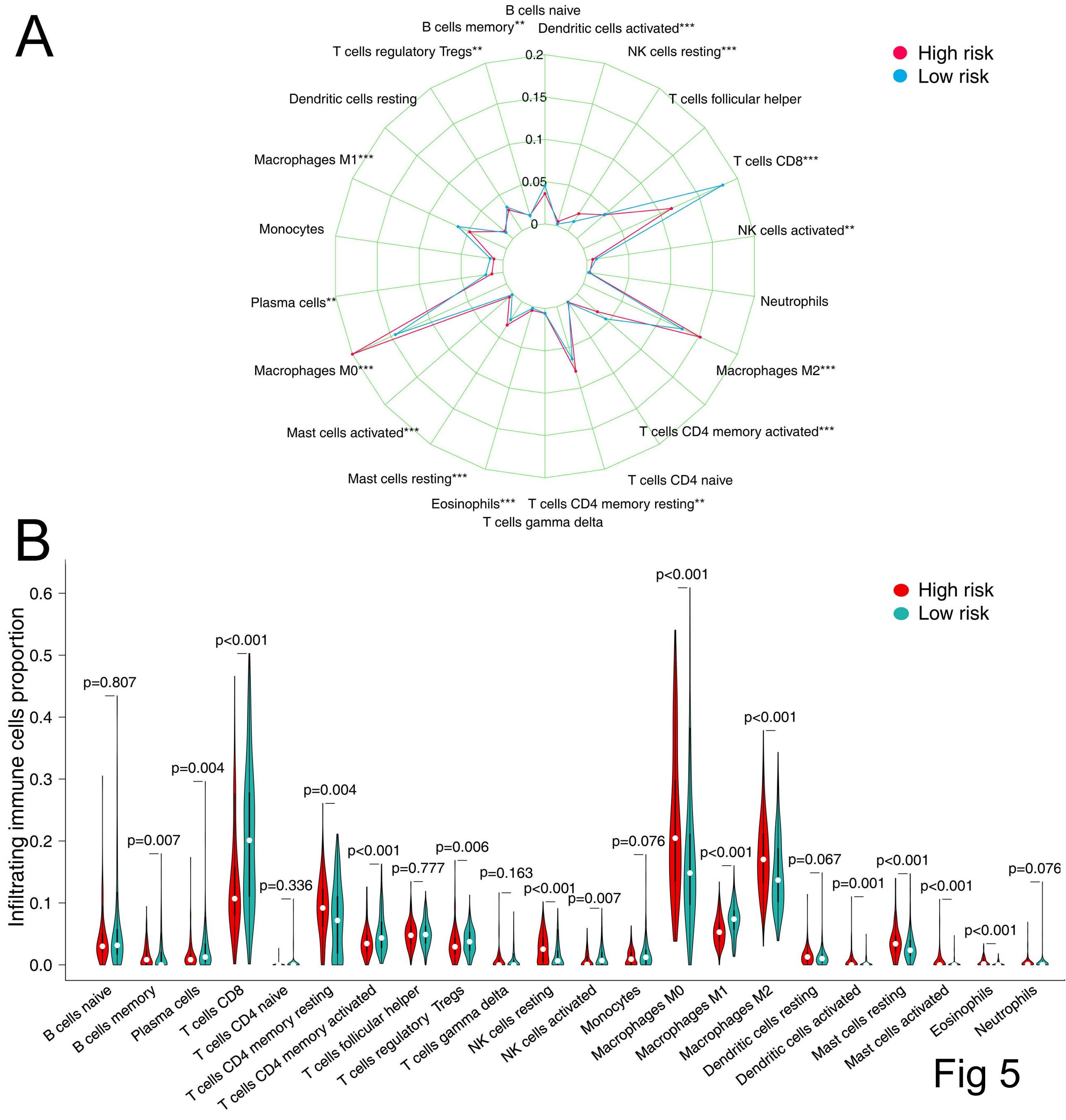
A
B cells naive
B cells memory**
Dendritic cells activated***
T cells regulatory Tregs**
NK cells resting***
Dendritic cells resting
T cells follicular helper
Macrophages M1***
T cells CD8***
Monocytes
NK cells activated**
Plasma cells**
Neutrophils
Macrophages M0***
Macrophages M2***
Mast cells activated***
T cells CD4 memory activated***
Mast cells resting***
T cells CD4 naive
Eosinophils***
T cells CD4 memory resting**
T cells gamma delta
0.2
0.15
0.1
0.05
0
High risk
Low risk
B
Infiltrating immune cells proportion
0.6
0.5
0.4
0.3
0.2
0.1
0.0
p=0.807
p=0.007
p=0.004
p<0.001
p=0.336
p=0.004
p<0.001
p=0.777
p=0.006
p=0.163
p<0.001
p=0.007
p=0.076
p<0.001
p<0.001
p<0.001
p=0.067
p=0.001
p<0.001
p<0.001
p<0.001
p=0.076
High risk
Low risk
B cells naive
B cells memory
Plasma cells
T cells CD8
T cells CD4 naive
T cells CD4 memory resting
T cells CD4 memory activated
T cells follicular helper
T cells regulatory Tregs
T cells gamma delta
NK cells resting
NK cells activated
Monocytes
Macrophages M0
Macrophages M1
Macrophages M2
Dendritic cells resting
Dendritic cells activated
Mast cells resting
Mast cells activated
Eosinophils
Neutrophils

Fig 5

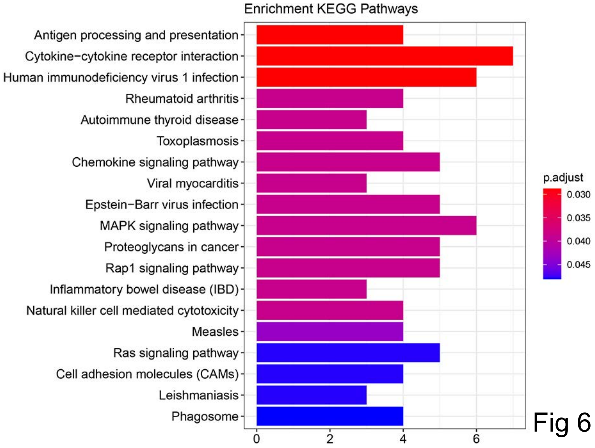
Enrichment KEGG Pathways
Antigen processing and presentation
Cytokine−cytokine receptor interaction
Human immunodeficiency virus 1 infection
Rheumatoid arthritis
Autoimmune thyroid disease
Toxoplasmosis
Chemokine signaling pathway
Viral myocarditis
Epstein−Barr virus infection
MAPK signaling pathway
Proteoglycans in cancer
Rap1 signaling pathway
Inflammatory bowel disease (IBD)
Natural killer cell mediated cytotoxicity
Measles
Ras signaling pathway
Cell adhesion molecules (CAMs)
Leishmaniasis
Phagosome
0
2
4
6
p.adjust
0.030
0.035
0.040
0.045

Fig 6

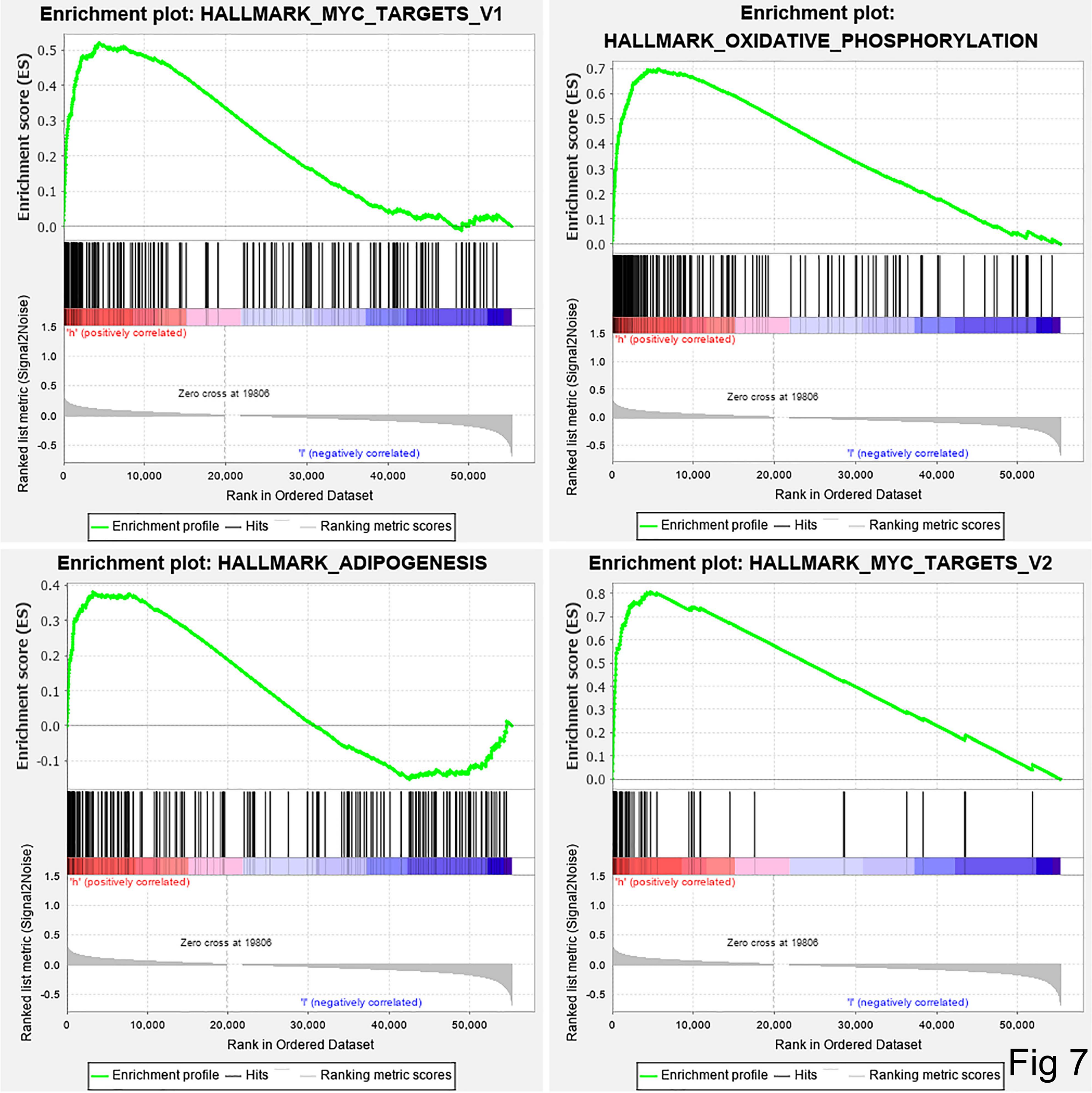

Enrichment plot: HALLMARK_MYC_TARGETS_V1
Enrichment plot: HALLMARK_OXIDATIVE_PHOSPHORYLATION
Enrichment plot: HALLMARK_ADIPOGENESIS
Enrichment plot: HALLMARK_MYC_TARGETS_V2
Enrichment score (ES)
Ranked list metric (Signal2Noise)
'h' (positively correlated)
Zero cross at 19806
'l' (negatively correlated)
Rank in Ordered Dataset
Enrichment profile
Hits
Ranking metric scores

Fig 7

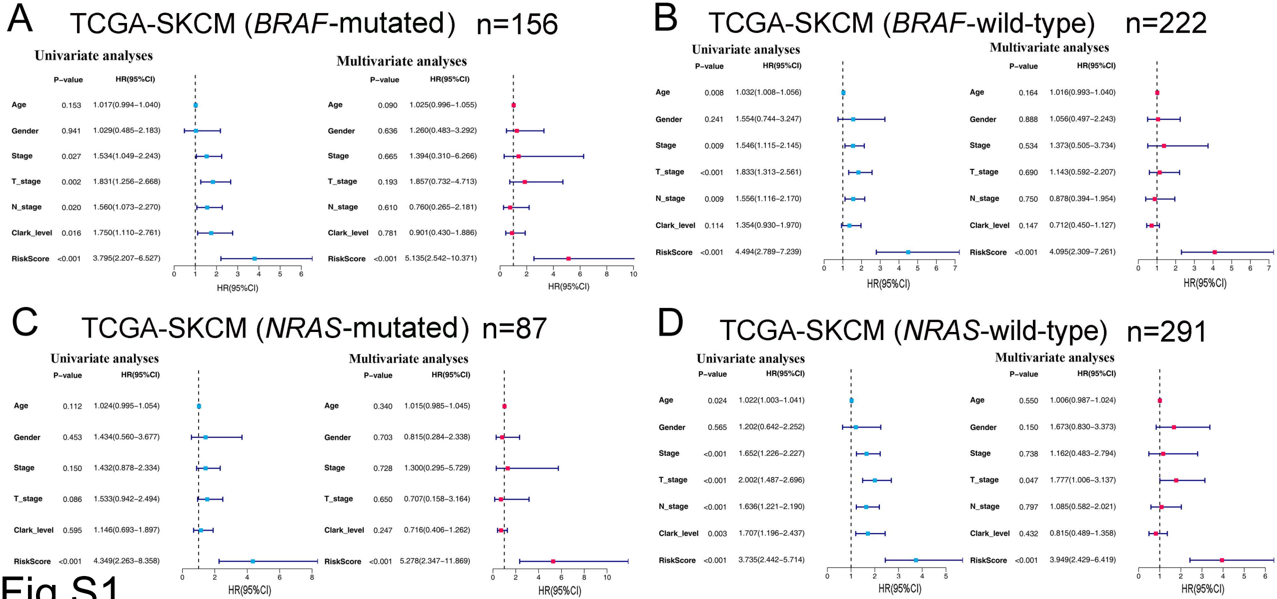
A
TCGA-SKCM (BRAF-mutated) n=156
Univariate analyses
P-value HR(95%CI)
Age 0.153 1.017(0.994-1.040)
Gender 0.941 1.029(0.485-2.183)
Stage 0.027 1.534(1.049-2.243)
T_stage 0.002 1.831(1.256-2.668)
N_stage 0.020 1.560(1.073-2.270)
Clark_level 0.016 1.750(1.110-2.761)
RiskScore <0.001 3.795(2.207-6.527)
HR(95%CI)
Multivariate analyses
P-value HR(95%CI)
Age 0.090 1.025(0.996-1.055)
Gender 0.636 1.260(0.483-3.292)
Stage 0.665 1.394(0.310-6.266)
T_stage 0.193 1.857(0.732-4.713)
N_stage 0.610 0.760(0.265-2.181)
Clark_level 0.781 0.901(0.430-1.886)
RiskScore <0.001 5.135(2.542-10.371)
HR(95%CI)
B
TCGA-SKCM (BRAF-wild-type) n=222
Univariate analyses
P-value HR(95%CI)
Age 0.008 1.032(1.008-1.056)
Gender 0.241 1.554(0.744-3.247)
Stage 0.009 1.546(1.115-2.145)
T_stage <0.001 1.833(1.313-2.561)
N_stage 0.009 1.556(1.116-2.170)
Clark_level 0.114 1.354(0.930-1.970)
RiskScore <0.001 4.494(2.789-7.239)
HR(95%CI)
Multivariate analyses
P-value HR(95%CI)
Age 0.164 1.016(0.993-1.040)
Gender 0.888 1.056(0.497-2.243)
Stage 0.534 1.373(0.505-3.734)
T_stage 0.690 1.143(0.592-2.207)
N_stage 0.750 0.878(0.394-1.954)
Clark_level 0.147 0.712(0.450-1.127)
RiskScore <0.001 4.095(2.309-7.261)
HR(95%CI)
C
TCGA-SKCM (NRAS-mutated) n=87
Univariate analyses
P-value HR(95%CI)
Age 0.112 1.024(0.995-1.054)
Gender 0.453 1.434(0.560-3.677)
Stage 0.150 1.432(0.878-2.334)
T_stage 0.086 1.533(0.942-2.494)
Clark_level 0.595 1.146(0.693-1.897)
RiskScore <0.001 4.349(2.263-8.358)
HR(95%CI)
Multivariate analyses
P-value HR(95%CI)
Age 0.340 1.015(0.985-1.045)
Gender 0.703 0.815(0.284-2.338)
Stage 0.728 1.300(0.295-5.729)
T_stage 0.650 0.707(0.158-3.164)
Clark_level 0.247 0.716(0.406-1.262)
RiskScore <0.001 5.278(2.347-11.869)
HR(95%CI)
D
TCGA-SKCM (NRAS-wild-type) n=291
Univariate analyses
P-value HR(95%CI)
Age 0.024 1.022(1.003-1.041)
Gender 0.565 1.202(0.642-2.252)
Stage <0.001 1.652(1.226-2.227)
T_stage <0.001 2.002(1.487-2.696)
N_stage <0.001 1.636(1.221-2.190)
Clark_level 0.003 1.707(1.196-2.437)
RiskScore <0.001 3.735(2.442-5.714)
HR(95%CI)
Multivariate analyses
P-value HR(95%CI)
Age 0.550 1.006(0.987-1.024)
Gender 0.150 1.673(0.830-3.373)
Stage 0.738 1.162(0.483-2.794)
T_stage 0.047 1.777(1.006-3.137)
N_stage 0.797 1.085(0.582-2.021)
Clark_level 0.432 0.815(0.489-1.358)
RiskScore <0.001 3.949(2.429-6.419)
HR(95%CI)

Fig S1
